# Urinary Albumin, Sodium, and Potassium and Cardiovascular Outcomes in the UK Biobank: Observational and Mendelian Randomization Analyses

**DOI:** 10.1161/HYPERTENSIONAHA.119.14028

**Published:** 2020-02-03

**Authors:** Daniela Zanetti, Helene Bergman, Stephen Burgess, Themistocles L. Assimes, Vivek Bhalla, Erik Ingelsson

**Affiliations:** 1 From the Division of Cardiovascular Medicine, Department of Medicine (D.Z., T.L.A., E.I.), Stanford University School of Medicine, CA; 2 Division of Nephrology, Department of Medicine (V.B.), Stanford University School of Medicine, CA; 3 Stanford Cardiovascular Institute (D.Z., T.L.A., E.I.), Stanford University, CA; 4 Stanford Diabetes Research Center (D.Z., E.I.), Stanford University, CA; 5 Department of Medical Epidemiology and Biostatistics, Karolinska Institute, Sweden (H.B.); 6 MRC Biostatistics Unit (S.B.), University of Cambridge, United Kingdom; 7 Department of Public Health and Primary Care (S.B.), University of Cambridge, United Kingdom; 8 Palo Alto VA Health Care System, CA (T.L.A.).

**Keywords:** blood pressure, cardiovascular diseases, coronary artery disease, heart failure, type 2 diabetes mellitus

## Abstract

Urinary biomarkers are associated with cardiovascular disease, but the nature of these associations is not well understood. We performed multivariable-adjusted regression models to assess associations of random spot measurements of the urine sodium-potassium ratio (UNa/UK) and urine albumin adjusted for creatinine with cardiovascular risk factors, cardiovascular disease, and type 2 diabetes mellitus (T2D) in 478 311 participants of the UK Biobank. Further, we assessed the causal relationships of these kidney biomarkers, used as proxies for kidney function, with cardiovascular outcomes using the 2-sample Mendelian randomization approach. In observational analyses, UNa/UK showed significant inverse associations with atrial fibrillation, coronary artery disease, ischemic stroke, lipid-lowering medication, and T2D. In contrast, urine albumin adjusted for creatinine showed significant positive associations with atrial fibrillation, coronary artery disease, heart failure, hemorrhagic stroke, lipid-lowering medication, and T2D. We found a positive association between UNa/UK and albumin with blood pressure (BP), as well as with adiposity-related measures. After correcting for potential horizontal pleiotropy, we found evidence of causal associations of UNa/UK and albumin with BP (β systolic BP ≥2.63; β diastolic BP ≥0.85 SD increase in BP per SD change in UNa/UK and urine albumin adjusted for creatinine; *P*≤0.04), and of albumin with T2D (odds ratio=1.33 per SD change in albumin, *P*=0.02). Our comprehensive study of urinary biomarkers performed using state-of-the-art analyses of causality mirror and extend findings from randomized interventional trials which have established UNa/UK as a risk factor for hypertension. In addition, we detect a causal feedback loop between albumin and hypertension, and our finding of a bidirectional causal association between albumin and T2D reflects the well-known nephropathy in T2D.

## Abstract 2

Supplemental Digital Content is available in the text.

**See related article, pp 625–627**

Cardiovascular disease (CVD) is a leading cause of mortality worldwide. In 2015, it accounted for 17.9 million deaths or almost one-third of all deaths globally.^1^ New strategies to prevent CVD are highly sought after from both a humanitarian and economical perspective. The identification of causal risk factors associated with CVD is expected to provide important insights on how to develop new strategies.

Over the past decades, several kidney-related biomarkers have been proposed to be associated with CVD,^2,3^ but their potential causal role in disease processes is not well understood. We used urinary biomarkers, as proxies for kidney function, to shed light on this relationship and to pinpoint causal risk biomarkers involved in this association.

The association of high sodium and low potassium with elevated blood pressure is supported by a large body of evidence in populations of different ancestry.^4–6^ The Prospective Urban Rural Epidemiology project recently assessed that in addition to hypertension, increasing potassium excretion is also associated with all major cardiovascular outcomes decrease. In the same study, urinary sodium showed association with CVD and strokes only in communities where mean intake was >5 g/d.^7^ In observational epidemiological studies, high albuminuria is associated with risk for cardiovascular events in individuals with or without diabetes mellitus.^8,9^ One recent study^10^ supported the existence of a bidirectional causal association between albuminuria and blood pressure, implying that albuminuria could increase risk of CVD through blood pressure. But there is a lack of prior studies comprehensively examining several urinary biomarkers reflecting different aspects of kidney function and their associations with blood pressure, type 2 diabetes mellitus (T2D), and CVD in a large study sample from the general population. Furthermore, the causal associations of these biomarkers with cardiometabolic traits are not well understood. We hypothesized that urinary excretion of albumin, sodium, and potassium are associated with cardiovascular and metabolic disease.

The aims of this study were to first determine the associations between urinary biomarkers, specifically urinary sodium, potassium, and albumin, with cardiovascular risk factors, T2D, and CVD in the UK Biobank by means of observational analyses including sex-stratified analyses; and then to test whether any of these associations are causal using a 2-sample Mendelian randomization (MR) approach, combining UK Biobank data with publicly available data from relevant genome-wide association studies (GWAS).

## Methods

### Data Availability

Datasets related to this article are available at UK Biobank resource (https://www.ukbiobank.ac.uk/). GWAS summary statistics for urinary biomarkers are available at GRASP resource (https://grasp.nhlbi.nih.gov/FullResults.aspx).

### Study Population

The UK Biobank is a longitudinal cohort study of >500 000 individuals aged 40 to 69 years initiated in the United Kingdom in 2006 to 2010.^11^ We used the data collected at the UK Biobank assessment centers at baseline, combined with information on incident events from the hospital and death registries. In our main analysis (N=478 311), we excluded participants with diagnoses indicating impaired kidney function (N=7221) and CVD at baseline (atrial fibrillation [AF], coronary artery disease [CAD], heart failure [HF], hemorrhagic stroke, or ischemic stroke [IS]; N=17 087; see below). We defined impaired kidney function as *International Classification of Diseases*, *Ninth Revision* (ICD-9) codes 581-589, 591, 2503, and V420; *Tenth Revision* (ICD-10) codes N00, N01, N03-N08, N10-N19, N25-29, E10.2, E11.2, E14.4, and Z99.2; and surgical codes for kidney (codes M01-M06, M08). In our sensitivity analysis (N=390 893), we additionally excluded participants using diuretics, ACE (angiotensin-converting enzyme) inhibitors, angiotensin II receptor blockers, or calcium channel blockers for any reason, and medications that are combination drugs including ≥1 of these categories (N=87 418), as these medications may influence glomerular filtration and electrolyte reabsorption and, therefore, urinary excretion of sodium, potassium, or albumin or may influence the effect of solute on CVD. Details of these measurements can be found in the UK Biobank Data Showcase (http://biobank.ctsu.ox.ac.uk/crystal/).

### Definition of Exposure and Cardiovascular Outcomes for Observational Analyses

The exposures of interest for our main analysis were urinary sodium (field ID 30530) to potassium (field ID 30520) excretion ratio (UNa/UK), and urinary albumin (field ID 30500) to creatinine (field ID 30510) ratio (UAlb/UCr). We analyzed urinary sodium to creatinine ratio (UNa/UCr) and urinary potassium to creatinine ratio (UK/UCr) for secondary analyses. Urine samples were collected at baseline in all UK Biobank participants. A random urinary spot was used as a measure of electrolyte excretion due to the difficulty in collecting and processing overnight or 24-hour urine samples in a very large population cohort with multiple study centers and a central biobank. All urinary biomarker measurements were performed on a single Beckman Coulter AU5400 clinical chemistry analyzer using the manufacturer’s reagents and calibrators, except for urinary albumin, which used reagents and calibrators sourced from Randox Bioscience. The Beckman Coulter analyzer used a photometric measurement for the determination of creatinine and albumin concentration and a potentiometric measurement for the determination of sodium and potassium concentration. The analysis method for urinary sodium and potassium involved a predilution of sample step, while for urinary albumin and creatinine assays it allowed samples with results exceeding the upper analytical limit of the assay to be diluted and reanalyzed. One advantage of using urine biomarkers in UK Biobank data is that all data were processed in the same laboratory with the same procedures. Internal quality control was performed for all the 4 urinary biomarkers data (http://biobank.ctsu.ox.ac.uk/crystal/docs/urine_assay.pdf). The method of using spot-urine samples to approximate 24-hour excretion is widely used, especially for surveys with large populations.^12,13^

Cardiovascular outcomes were defined using the inpatient hospital and death registries, including primary and secondary causes to maximize power. AF was defined as ICD-9 code 427.3, ICD-10 code I48, and surgical codes K50.1, K62.2-K62.4. CAD was defined as ICD-9 codes 410-411, ICD-10 codes I20.0, I21, and I22; and surgical codes for percutaneous transluminal coronary angioplasty and coronary artery bypass graft (codes K40-K46, K49-K50, and K75). HF was defined as ICD-9 code 428 and ICD-10 code I50. Stroke was defined as hemorrhagic (ICD-9: 430-432, ICD-10: I60-I62) or IS (ICD-9: 433-434, ICD-10: I63). The hospital registry-based follow-up ended on March 31, 2015 in England; August 31, 2014 in Scotland; and February 28, 2015 in Wales. We censored individuals either on these dates, at the time of event in question, or at the time of death, whichever occurred first. The death registry included all deaths that occurred before January 31, 2016 in England and Wales, and November 30, 2015 in Scotland.

### Definition of Confounders for Observational Analyses

We used data from questionnaires to derive the following potential confounders: sex (ID 31), age (ID 21003), region of the UK Biobank assessment center (ID 54; recoded to 3 countries: United Kingdom, Scotland, and Wales), ethnicity (ID 21000; recoded to 4 groups: black, Asian, white, and mixed), smoking status (ID 20116, recoded to 3 groups: never, previous, and current), alcohol use (ID 100022, weekly alcohol intake in grams), degree of physical activity (ID 894, recoded to 2 groups: days/wk of moderate physical activity <5, days/wk of moderate physical activity ≥5), and a Townsend index reflecting socioeconomic status (ID 189). Physical measurements were used to define systolic blood pressure (SBP; ID 4080, but if missing ID 93), diastolic blood pressure (DBP; ID 4079, but if missing ID 940), body fat percentage (ID 23099), body mass index (BMI; ID 21001), and waist-to-hip ratio (WHR; ID 48/ID 49). Lipid medications (ID 20003; including the following medications: simvastatin, pravastatin, fluvastatin, atorvastatin, rosuvastatin, ezetimibe, nicotinic acid product, or fenofibrate) were used as a proxy for hyperlipidemia, as lipid level measurements were not available in UK Biobank at the time of the present study. T2D was defined as having a diagnosis of ICD-9 code 250.10 or 250.12, or ICD-10 code E11 in the inpatient hospital register; diabetes mellitus diagnosed by a physician (ID 2443) after 35 years old (ID 2976), or being treated with antidiabetic medication, but without insulin treatment in the first year (ID 2986).

### Statistical Methods

#### Observational Analysis

Multivariable-adjusted Cox proportional hazards models were performed to determine associations of our exposures with AF, CAD, HF, hemorrhagic stroke and IS events, separately; during a median follow-up time of 6.1 years. We performed multivariable linear regression models to determine associations of exposures with SBP, DBP, body fat percentage, BMI, and WHR and multivariable logistic regression models to study associations of urinary biomarkers with lipid medications and T2D. We assessed evidence of nonlinear effects of UNa/UK and UAlb/UCr on different outcomes using spline regression models. We use the DAGitty web tool (http://dagitty.net/dags.html) to systematically construct our multivariable model adjusting for confounders. All association analyses were adjusted for age, sex, region of the UK Biobank assessment center, ethnicity, smoking, alcohol, physical activity, Townsend index, blood pressure (DBP and SBP), obesity (BMI, body fat percentage, WHR), lipid medications, T2D, and medications affecting renal excretion. In addition, we performed secondary analyses for UNa/UK without adjustment for blood pressure, to further disentangle a possible mediating or confounding effect of blood pressure on CVD, lipids, T2D and obesity traits; and adjusting our models only for age, sex, region of the UK Biobank assessment center, and ethnicity (minimally adjusted model). Further, we performed sex-stratified analyses to study sex differences of these associations. A Bonferroni-corrected threshold of 4.17×10^−3^ (adjusting for 12 comparisons) was used to adjust for multiple testing to avoid false-positive findings. Cox regressions analyses were conducted with the R package Survival (version 3.3.0).

#### Mendelian Randomization

MR uses genetic variants as instrumental variables to make inferences about causal effects based on observational data. Associations between modifiable exposures and disease seen in observational epidemiology are often prone to reverse causation and confounding, and thus noninformative with regards to causality. MR—based on the random assortment of genes from parents to offspring that occurs at conception—provides a method for assessing the causal nature of associations between exposures (risk factors, biomarkers) and outcomes. Unlike environmental exposures, genetic variants cannot change as a result of the outcome (hence, excluding reverse causation) and are not generally associated with the wide range of behavioral, social, or physiological factors that confound classic observational associations. This means that if a genetic variant is used as a proxy for an environmentally modifiable exposure or biomarker, it is unlikely to be affected by reverse causation or confounding in the way that direct measures of the exposure or biomarker will be.^14^

We performed 2-sample MR analyses using as outcomes data from publicly available consortia, except for blood pressure where we performed a GWAS in UK Biobank (as the publicly available GWAS summary statistics were adjusted for BMI).

We previously performed GWAS of all the urinary biomarkers in up to 327 616 unrelated Europeans participants of the UK Biobank.^15^ We excluded individuals who had withdrawn consent at the time of this study, who were related, and those who did not self-report as white or did not cluster with Europeans based on principal component analysis of genetic data. We adjusted all models for age, sex, batch (3 levels; UK Biobank Lung Exome Variant Evaluation, UK Biobank release 1, and UK Biobank release 2), and the first 10 genotype principal components, and restricted association analyses to single-nucleotide polymorphisms (SNPs) with minor allele count ≥30 and imputation quality information score (info) ≥0.8. We then used genome-wide significant independent hits (after linkage disequilibrium clumping using a window of 500 kb and *r*^2^ cutoff=0.05) associated with UNa/UK, UNa/UCr, UK/UCr, and UAlb/UCr as instrument variables for the MR analyses. The results of this previous GWAS^15^ are available at GRASP resource (https://grasp.nhlbi.nih.gov/FullResults.aspx). A list of the variants included in the instrument variable is shown in Table S1 in the online-only Data Supplement. Finally, as a last step before performing the MR analyses, we performed several data harmonization steps. Since, the effects of a SNP on the exposure and the outcome must correspond to the same allele, we identified variants with unmatched effect alleles and inferred the forward strand allele using allele frequency information. After that, we flipped their effect estimates and the effect allele frequencies in one of the data sets.

We assessed the causal relationships of the 4 urinary biomarkers with risk factors for CVD (SBP, DBP, BMI, and WHR). We did not study causal associations of UNa/UK, UNa/Cr, and UK/Cr with any hard CVD end points due to lack of statistical power (Table S2). We assessed the causal relationships of UAlb/UCr with AF and T2D (power to detect causal effects >75%; Table S2).

We performed 2-sample MR using 3 separate methods to estimate causal effects: the standard inverse variance weighted regression; as well as 2 robust regression methods, the weighted median-based method, and Egger regression.^16^ We performed leave-one-out sensitivity analyses to identify if a single SNP was driving an association. In addition, we performed bidirectional MR and multivariable MR for significant causal outcomes.

We performed the 2-sample MR analyses,^16,17^ as well as the bidirectional MR and the multivariable MR with the R package, 2-sample MR. In addition, we used the Mendelian Randomization Pleiotropy RESidual Sum and Outlier software^18^ to minimize the risk of horizontal pleiotropy affecting our results. A fundamental assumption of MR is the lack of horizontal pleiotropy assumption which requires that the genetic variants used for MR analyses act on the outcome exclusively through the exposure of interest (if there is an association). Horizontal pleiotropy occurs when the variant has an effect on other traits outside of the pathway of the exposure of interest that has an impact on the target outcome. As a violation of the lack of horizontal pleiotropy assumption, horizontal pleiotropy can distort MR tests, leading to inaccurate causal estimates, loss of statistical power, and potential false-positive causal relationships. It should be noted that vertical pleiotropy or mediation (genetic variants associated with the exposure and several other steps along the causal pathway before the outcome) is not a violation of any MR assumptions (and a common feature in biology). We applied 2 robust methods with different assumptions about the behavior of pleiotropic variants: (1) MR-Egger,^19^ which assumes that the INstrument Strength is Independent of the Direct Effect, which means that pleiotropic effects are independent of phenotypic effects across variants and (2) Mendelian Randomization Pleiotropy RESidual Sum and Outlier,^18^ that excludes outlying variants as being potentially pleiotropic.

Details of the GWAS summary statistics used to performed MR analyses and variance explained by our instruments can be found in Table S2.

We estimated statistical power for each of the MR analyses using variance explained from our exposures and effect size from observational analyses and an alpha threshold of 0.05. In addition, we calculated the statistical power using a fixed effect of 1.15 for binary trait and 0.15 for continuous traits. Power for MR analyses was estimated with the online tool at https://sb452.shinyapps.io/power/.

## Results

Baseline characteristics of UK Biobank participants are shown in Table 1. In the main analysis, the mean age at baseline was 56.3 years (SD, 8.1 years), and 56% of participants were females. During a median follow-up time of 6.1 years, 22 212 incident CVD cases occurred in participants free from the disease at baseline (9196 AF; 7375 CAD; 2775 HF; 978 hemorrhagic stroke; and 1888 IS events; Table 2).

**Table 1.**
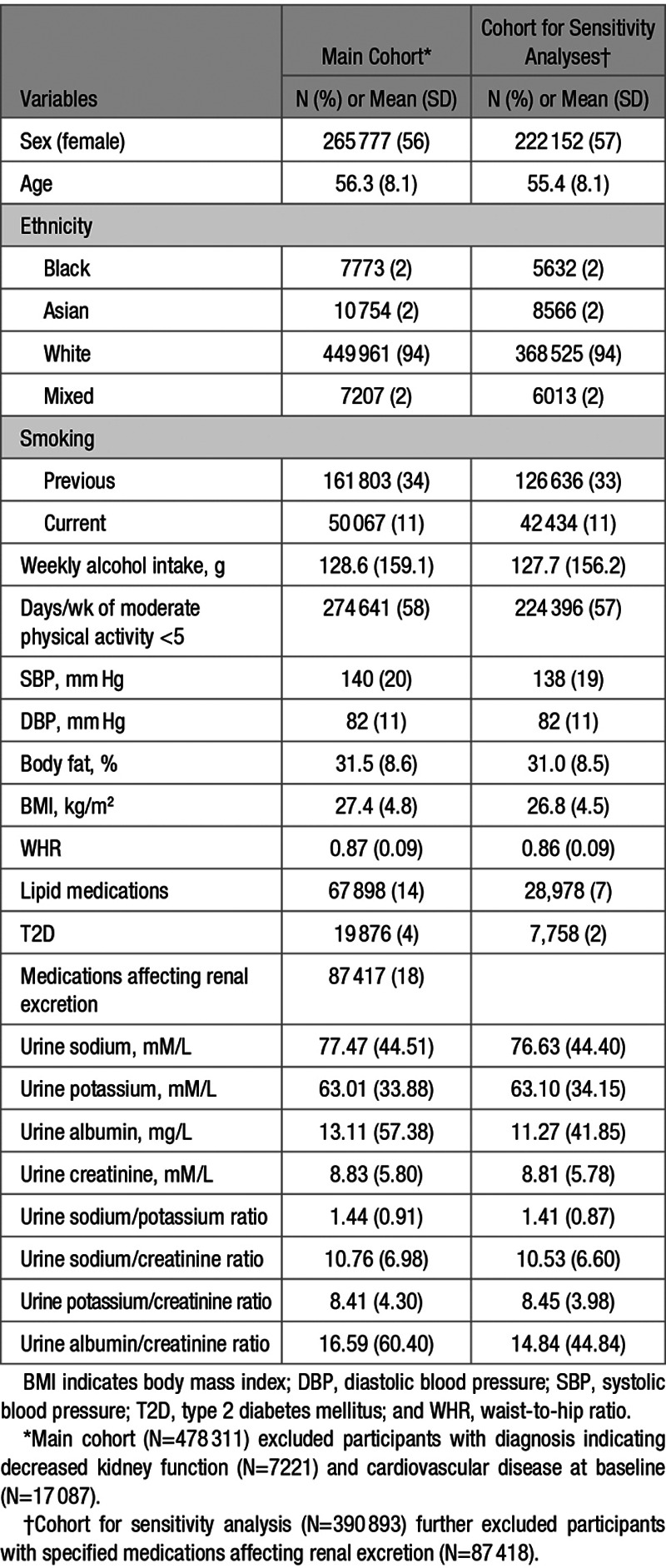
Baseline Characteristics of UK Biobank Participants for Main (N=478 311) and Sensitivity (N=390 893) Analyses

**Table 2.**
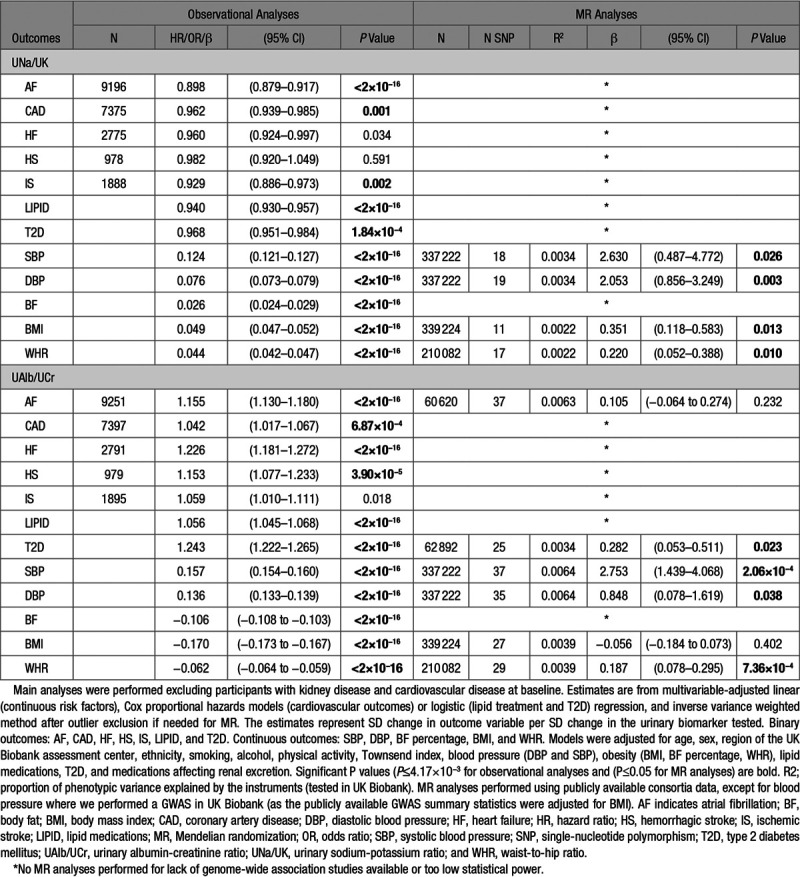
Observational and MR Analyses of Associations of UNa/UK and UAlb/UCr With Cardiovascular Outcomes

### Observational Analyses

Table 2 summarizes the results from our main observational analyses (full results in Table S3). UNa/UK showed significant inverse associations (ie, higher UNa/UK associated with lower disease risk) with AF, CAD, lipid-lowering medication, and T2D. In contrast, higher UNa/UK was associated with higher SBP and DBP, as well as increased body fat percentage, BMI, and WHR (*P*≤0.0042). When we performed these associations using sodium and potassium adjusted for creatinine, we detected similar and consistent results for UNa/UCr, but slightly different results for UK/UCr. Indeed, higher UK/UCr was associated with higher lipid-lowering medication and T2D and lower SBP (Table S3 and Figure S1). When we excluded blood pressure from our multivariable-adjusted models, we observed mostly similar results, except the associations of UNa/UK with CAD and IS that were attenuated (Table S4A). In our minimally adjusted models (adjusted only for age, sex, region of the UK Biobank assessment center, and ethnicity), we observed a significant inverse and direct association of UNa/UK with AF and CAD, respectively (Table S4B).

UAlb/UCr showed significant positive associations with AF, CAD, HF, hemorrhagic stroke, lipid-lowering medication, and T2D. Further, high UAlb/UCr was associated with higher SBP and DBP. In contrast, UAlb/UCr showed significant inverse associations with body fat percentage, BMI, and WHR. The negative association with obesity traits (body fat percentage, BMI, and WHR) was consistent also for UNa/UCr and UK/UCr (Table S3) and was driven by the adjustment for creatinine. Indeed, urine sodium, potassium, and albumin not adjusted for creatinine showed significant positive associations with obesity traits (Table S5).

In our sensitivity analyses, after excluding participants using diuretics, ACE inhibitors, angiotensin II receptor blockers, or calcium channel blockers, we observed consistent results. For a few associations, UNa/UK with CAD, IS, and T2D; UNa/UCr and UK/UCr with HF; and UAlb/UCr with CAD, we observed consistent directions with the main analyses, without reaching significance, probably due to the lower sample size (lower statistical power; Table S3 and Figure S1).

We excluded nonlinear associations between UNa/UK and UAlb/UCr and all outcomes tested (*P*>0.05), except for HF (*P*=9.0×10^-8^, UNa/UK) and IS (*P*=0.001, UAlb/UCr) by spline regression (Figure S2 and S3).

When participants eligible for inclusion in the main analysis were stratified by sex (Table S6 and Figure S4), no additional significant associations were found between exposures and outcomes in either subset. All associations between urinary biomarkers and outcomes remained significant and consistent with the main analyses in both men and women. Events were more common in the male sample set for all CVD outcomes (Table S6). Generally, males displayed larger effect estimates than females. For disease outcomes, this potentially could be explained by better statistical power (more events), while the power should be equal for continuous traits (as the number of measurements were similar). The strongest sex interactions were observed for UAlb/UCr and T2D (*P*<2×10^−16^) and across urinary biomarkers and obesity traits (*P*<2×10^−16^; Table S6 and Figure S4).

### Mendelian Randomization

After correcting for horizontal pleiotropy, we found evidence of causal associations between UNa/UK and BMI, UAlb/UCr and T2D, and of both biomarkers (UNa/UK and UAlb/UCr) with blood pressure and WHR (Table 2 and Figure 1). A leave-one out sensitivity analysis did not highlight any SNPs with a large effect on the results. After excluding heterogeneous SNPs using Mendelian Randomization Pleiotropy RESidual Sum and Outlier (Figures S5 through S8), our analysis showed no significant heterogeneity and no significant directional horizontal pleiotropy. Numbers of variants included in the analyses, number of outliers excluded, and full results are shown in Table S7. We only performed MR analyses for outcomes for which we had at least 75% statistical power (Table S2).

**Figure.**
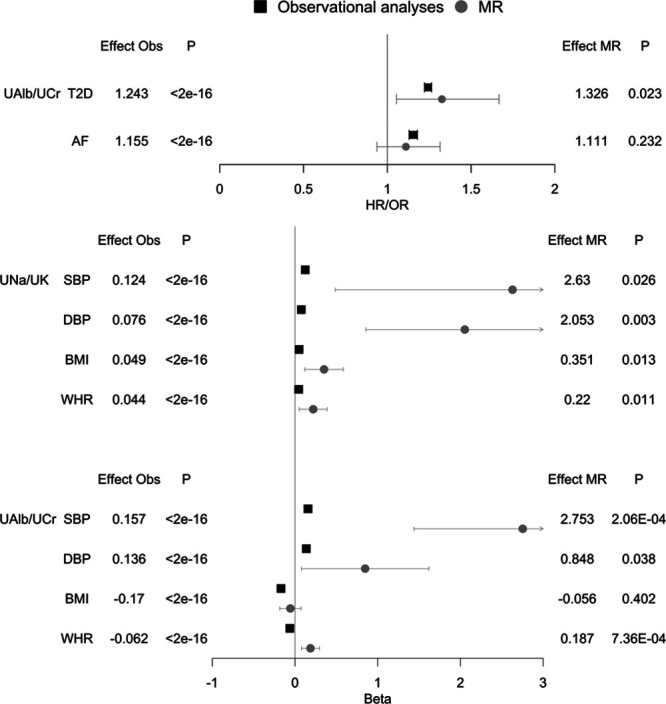
Observational and Mendelian randomization (MR) analyses of urinary sodium-potassium ratio (UNa/UK) and urinary albumin-creatinine ratio (UAlb/UCr) with cardiovascular outcomes in UK Biobank. Binary outcomes: type 2 diabetes mellitus (T2D) and atrial fibrillation (AF). Continuous outcomes: systolic and diastolic blood pressure (SBP and DBP), body mass index (BMI), and waist-to-hip ratio (WHR). Main analysis (N=478 311) excluding participants with diagnoses indicating decreased kidney function (N=7221) and cardiovascular disease at baseline (N=17 087). Associations were performed using multivariable-adjusted linear, logistic, and Cox proportional hazards models in main observational analyses and inverse variance weighted method for MR. The betas from linear regression represent SD change in outcome variable per SD change in urinary sodium and potassium. The hazard and odds ratios are given per SD change in urinary sodium and potassium. Model adjustment: age, sex, region of the UK Biobank assessment center, ethnicity, smoking, alcohol, physical activity, Townsend index, blood pressure (DBP and SBP), obesity (BMI, body fat percentage, WHR), lipid medications, T2D, and medications affecting renal excretion. MR analyses performed using publicly available consortia data, except for blood pressure where we performed a genome-wide association studies (GWAS) in UK Biobank (as the publicly available GWAS summary statistics were adjusted for BMI). HR indicates hazard ratio; and OR, odds ratio.

We found evidence of causal bidirectional effect across UNa/UK and UAlb/UCr and blood pressure, and between albumin and T2D (Table S8).

We performed multivariable MR using all established GWAS significant variants for UAlb/UCr, SBP, and WHR as predictor variables and GWAS of T2D and AF as outcome variables. We detected an independent association between UAlb/UCr and T2D, while the association of UAlb/UCr with AF was mediated by SBP (Table S9).

## Discussion

### Principal Findings

We studied associations of urinary biomarkers, used as proxies for kidney function, with cardiometabolic disease in 478 311 individuals free of chronic kidney disease and CVD at baseline. We made 4 main findings. First, we found a positive association between UNa/UK and UAlb/UCr with blood pressure, as well as with adiposity-related measures (body fat percentage, BMI, or WHR). Second, we observed a direct association of UAlb/UCr with CVD incidence but an inverse association of UNa/UK with incident CVD and T2D in traditional observational analyses. Third, the strongest sex differences were observed for associations between UAlb/UCr and T2D, and across urinary biomarkers and obesity traits. However, all associations were directionally consistent. Lastly, using a MR approach, we provided evidence that higher UNa/UK is causally related with higher blood pressure, and we highlighted a causal feedback loop between albumin and hypertension and between albumin and T2D.

### Comparison With Prior Observational Studies

Our results are consistent with previous literature that reported that high UAlb/UCr is associated with higher susceptibility to CVD^8^ and hypertension.^10^

Previous studies have shown inconsistent results between sodium excretion and the risk of CVD. Welsh et al^22^ did not detect significant associations between quintile of sodium excretion and cardiovascular outcomes in the UK Biobank. In contrast, our findings from observational analyses show an inverse association between UNa/UK excretion and AF, CAD, and IS. This discrepancy is likely due to different statistical modeling, specifically that they used the Kawasaki formula to convert spot sodium and potassium measurements into estimated 24-hour excretion; that they analyzed quintiles of excretion rather than continuous variables; and that they adjusted their models for a different set of covariates compared with the ones used in our study. Indeed, exploratory analyses indicated a positive effect of UNa/UK on CAD risk when we adjusted our model only for sex, age, center, and ethnicity (Table S4B).

A recent observational study reported a positive association between sodium excretion and stroke only in communities where mean sodium intake was >5 g/d, an association largely confined to China. By contrast, they found an inverse relation with myocardial infarction and mortality.^7^ The discrepancies between our study and this prior study are likely to be explained by different study populations (Chinese versus British), baseline salt intake, different statistical approaches (linear versus categorical variables, adjustment for covariates).

We detected an inverse association between UNa/UK and hyperlipidemia in line with a previous meta-analysis of randomized controlled trials which found that a low sodium was associated with an increase in cholesterol and triglycerides.^20^ We also identified an inverse association between UNa/UK excretion and T2D in the UK Biobank cohort. To the best of our knowledge, the relationship between UNa/UK and incidence of T2D has not been previously described. In contrast, the association between urine albumin and diabetes mellitus was already observed in previous studies,^21^ and it is consistent with the positive association detected by our study.

In addition, we also confirmed the well-established direct association between sodium and blood pressure^5,6,22^ (using urinary sodium adjusted for creatinine), as well as the inverse association between potassium and blood pressure^5,6,23–25^ (using urinary potassium adjusted for creatinine) in the UK Biobank cohort. Regarding UAlb/UCr and blood pressure, our results are consistent with a recent previous study^10^ which supported the existence of a bidirectional causal association between albuminuria and blood pressure.

We observed positive associations between UNa/UK and UAlb/UCr with obesity and adiposity-related risk factors (body fat percentage, BMI, and WHR). These results are consistent with previous studies that have suggested albuminuria^26^ and sodium^27,28^ as independent risk factor for obesity. One possibility for this association would be that it reflects higher intake of sugar-sweetened soft drinks (resulting in increased sodium excretion and at the same time, obesity). However, there are also other potential physiological explanations for why high sodium excretion could contribute to obesity, independently of energy intake or soft drink consumption.^27^ One such possible mechanism includes direct effects of sodium on adipose tissue, supported by the observation that rats fed a high-salt diet had a higher levels of plasma leptin (presumably reflecting fat mass), as well as excessive accumulation of white adipose fat compared with rats with lower salt intake.^29^ Further, another recent study in mice showed that high intake of salt activates the aldose reductase-fructokinase pathway in the liver and hypothalamus, leading to endogenous fructose production with the development of leptin resistance and hyperphagia that cause obesity, insulin resistance, and fatty liver.^30^

Our MR results mirror and extend findings from previous randomized interventional trials^23,24^ that have established sodium as a risk factor for hypertension. In addition, we highlight a causal feedback loop between albumin and T2D, and we also replicate the existence of a bidirectional causal association between albuminuria and blood pressure that was reported by a recent MR study.^10^

### The Potential Causal Role of Urinary Biomarkers in Blood Pressure and T2D

The incidence and prevalence of hypertension continue to rise, presumably due to an aging population, increasing obesity, and physical inactivity.^31^ Substantial evidence from clinical trials have demonstrated that high sodium and low potassium are significantly associated with elevated blood pressure.^23,24^ Previous studies also show that reducing dietary sodium in individuals with prehypertension decreases the risk of cardiovascular events and overall mortality.^32–34^

In this study, we explore the association of urinary biomarkers with cardiometabolic outcomes using genetic data by means of a MR approach. We confirm a causal role of high sodium and low potassium excretion in higher susceptibility for increased blood pressure. Endothelial dysfunction probably plays an important role to modulate the influence of high sodium on blood pressure, although the exact mechanisms remain elusive.^35^

We also observe a bidirectional causal association of albumin with blood pressure. The causal association of albumin with blood pressure was already highlighted in a recent study that suggested the existence of a feed-forward loop where elevated blood pressure leads to increased albuminuria, which in turn further increases blood pressure.^10^ The presence of albuminuria is a powerful predictor of renal and cardiovascular risk in patients with T2D and hypertension. Multiple studies have shown that decreasing albuminuria reduces the risk of adverse renal and cardiovascular outcomes. The pathophysiology is not definitively known, but also in this case, it is hypothesized to be related to endothelial dysfunction, inflammation, and abnormalities in the renin-angiotensin-aldosterone system.^36^

Glomerular endothelial dysfunction is also implicated in the link between albuminuria and T2D.^37,38^ Our data suggest an independent causal association between T2D and albumin not mediated by blood pressure. Urine albumin arises primarily from the increased passage of albumin through the glomerular filtration barrier that is insufficiently reabsorbed by tubular epithelium. The filtration barrier is comprised of the endothelium, glomerular basement membrane, and podocytes. T2D can lead to disruption of each of these components including the endothelium in which disruption of the endothelial glycocalyx through dysregulation by the diabetic milieu.^37^ Indeed, diabetic patients have decreased systemic glycocalyx volume, and this is correlated with the presence of albuminuria.^39^

### Strengths and Limitations

Our study is the largest and most comprehensive study of causal associations of urinary biomarkers with cardiovascular risk factors, T2D, and CVD to date. Strengths of our study include the large sample size, the robustness of our findings, the most recent and powerful GWAS summary statistics as outcomes, and several sensitivities analyses to decrease the risk of pleiotropy.

Our study also has several limitations. First, we were limited to using measures available in the UK Biobank. As a result, we used random spot measurements of urine samples, although multiple day 24-hour urine collection is recommended as the gold standard method for assessing sodium intake.^40^ The stochasticity of these spot urine samples may lower statistical power due to the introduction of random variation; however, it is unlikely to introduce systematic biases causing false positives but rather drive associations towards the null. Second, the UK Biobank did not collect detailed information about the urine sample collections. Consequently, we do not have information about the time of the day when they were collected and the diet of the individuals before collection. Third, the vast majority of participants were of European ancestry despite the inclusion of several non-European ethnicities. Hence, our results may not be generalizable to other race/ethnic groups with significantly different diets, prevalence and predispositions to cardiometabolic disease. Finally, statistical power to detect potentially causal relationships through our MR studies was limited for some traits, at least for smaller effects, including some of those observed in our traditional epidemiological analyses.

### Conclusions

Our comprehensive study of urinary biomarkers performed using state-of-the-art analyses of causality mirrors and extends findings from randomized interventional trials which have established UNa/UK as a risk factor for hypertension. In addition, we detect a causal feedback loop between albumin and hypertension, and our finding of a bidirectional causal association between albumin and T2D reflects the well-known nephropathy in T2D.

### Perspectives

#### Causal Association of Urinary Biomarkers and Cardiometabolic Outcomes

Urine biomarkers related with kidney function are strongly associated with several common diseases including CVD and diabetes mellitus, but it is unknown whether these associations are causal.

Our results indicate that higher UNa/UK, used as proxy for kidney function, is causally related with higher blood pressure. We highlight a causal feedback loop between UAlb/UCr and hypertension and between UAlb/UCr and T2D. In addition, our results indicate that the causal association between T2D and urinary albumin is not mediated by blood pressure.

The knowledge about the causality of these associations arising from our work may shed light on pathophysiological mechanisms underlying the development of CVD. These results improve the biological understanding of the connection between kidney function and CVD and point to new therapeutic strategies to prevent common diseases.

## Acknowledgments

This research has been conducted using the UK Biobank Resource under Application Number 13721. D. Zanetti, H. Bergman, E. Ingelsson contributed susbtantially to the conception and design, acquisition of data, or analysis and interpretation of data; D. Zanetti, S. Burgess, T.L. Assimes, V. Bhalla, and E. Ingelsson contributed in drafting the article or revising it critically for important intellectual content; D. Zanetti, H. Bergman, S. Burgess, T.L. Assimes, V. Bhalla, and E. Ingelsson contributed in final approval of the version to be published and; D. Zanetti, H. Bergman, S. Burgess, T.L. Assimes, V. Bhalla, and E. Ingelsson contributed in agreement to be accountable for all aspects of the work.

## Sources of Funding

The research was performed with support from National Institutes of Health (1R01HL135313-01; 1R01DK106236-01A1) and the Stanford Diabetes Research center award (P30DK116074). D. Zanetti was supported by the American Heart Association Postdoctoral Fellowship (19POST34370115). S. Burgess was supported by Sir Henry Dale Fellowship jointly funded by the Welcome Trust and the Royal Society (grant number 204623/Z/16/Z).

## Disclosures

E. Ingelsson is a scientific advisor for Precision Wellness for work unrelated to the present project. The other authors report no conflicts.

## Supplementary Material



## Novelty and Significance

### What Is New?

- Using the largest sample sizes to date for exposures and outcomes and several sensitivities analyses to minimize the risk of pleiotropy, we investigated the potential causal role of urinary biomarkers, used as proxies for kidney function, for development of hypertension, and other cardiovascular traits.

### What Is Relevant?

- Our study mirrors and extends findings from randomized interventional trials which have established urine sodium-potassium ratio as a risk factor for hypertension. In addition, our findings suggest causal feedback loops between urinary albumin and hypertension, and urinary albumin and type 2 diabetes mellitus.

### Summary

Our study improves the biological understanding of connections between kidney function and hypertension and other cardiovascular traits and point to new therapeutic strategies to prevent these common diseases.
