# Supplementary figures and images for: Urinary Albumin, Sodium, and Potassium and Cardiovascular Outcomes in the UK Biobank: Observational and Mendelian Randomization Analyses
Source: Hypertension. 2020 Feb 3;75(3):714–22. doi: 10.1161/HYPERTENSIONAHA.119.14028 (PMC8032218; doi:10.1161/HYPERTENSIONAHA.119.14028)

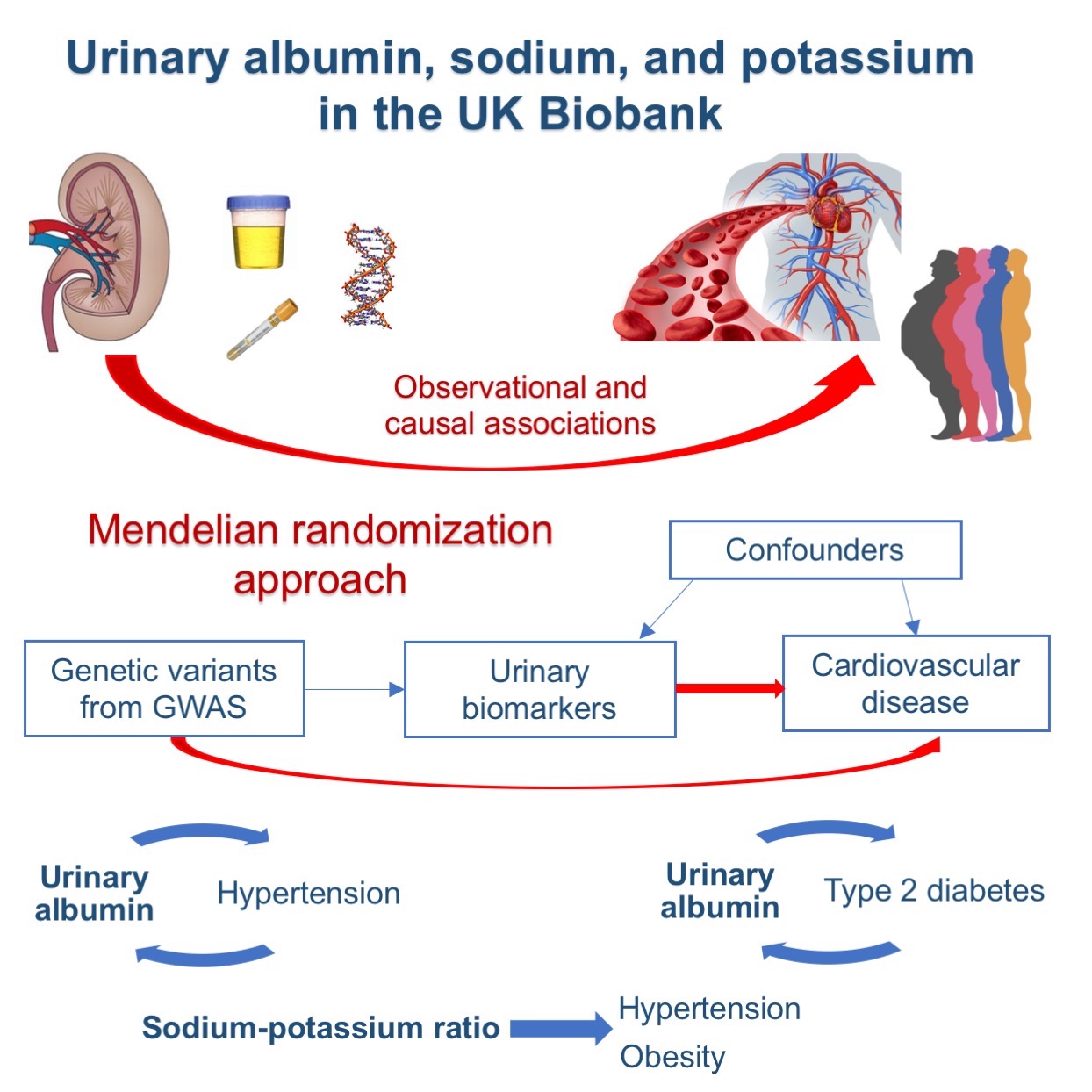

Supplement: Supplementary file 1 [file hyp-75-714-s001.jpg]
