# Supplementary material for: Urinary Albumin, Sodium, and Potassium and Cardiovascular Outcomes in the UK Biobank: Observational and Mendelian Randomization Analyses
Source: Hypertension. 2020 Feb 3;75(3):714–22. doi: 10.1161/HYPERTENSIONAHA.119.14028 (PMC8032218; doi:10.1161/HYPERTENSIONAHA.119.14028)
Supplement: Supplementary file 2 [file hyp-75-714-s002.pdf]

Supplemental Material

Urinary albumin, sodium, and potassium and cardiovascular outcomes in the UK Biobank: observational and Mendelian randomization analyses

Running Title: Urinary biomarkers in the UK Biobank

Daniela Zanetti[1,2,3], PhD; Helene Bergman[4], BSc; Stephen Burgess[5], PhD; Themistocles L. Assimes[1,2,6], MD; Vivek Bhalla[7], MD; Erik Ingelsson[1,2,3], MD, PhD

1. Department of Medicine, Division of Cardiovascular Medicine, Stanford University School of Medicine, Stanford, CA;
2. Stanford Cardiovascular Institute, Stanford University, Stanford, CA;
3. Stanford Diabetes Research Center, Stanford University, Stanford, CA;
4. Department of Medical Epidemiology and Biostatistics, Karolinska Institute, Sweden;
5. Department of Public Health and Primary Care, University of Cambridge, Cambridge, UK;
6. Palo Alto VA Health Care System, Palo Alto, CA;
7. Department of Medicine, Division of Nephrology, Stanford University School of Medicine, Stanford, CA.

Address for Correspondence:
Erik Ingelsson, MD, PhD, FAHA
Falk Cardiovascular Research Center, CV154
300 Pasteur Drive, Mail code: 5406; Stanford, CA 94305; USA
Phone: +1-650-656-0089; E-mail: eriking@stanford.edu

Supplemental Tables

Table S1. Variants included in the instrument variable for Mendelian randomization analyses.

UNa/UK						
**SNP**	**EA**	**OA**	**EAF**	**BETA**	**SE**	**P**
rs1194277	G	C	0.508	0.015	0.002	1.57E-10
rs7157772	T	G	0.612	0.015	0.002	4.66E-10
rs299938	G	A	0.234	-0.017	0.003	2.13E-09
rs8096658	G	C	0.488	-0.014	0.002	2.69E-08
rs2903625	T	A	0.384	0.014	0.002	2.41E-08
rs34783010	T	G	0.193	-0.022	0.003	7.07E-13
rs1698114	G	A	0.484	0.016	0.003	1.39E-09
rs6754311	C	T	0.248	0.017	0.003	1.03E-09
rs3770435	C	T	0.362	0.017	0.003	3.06E-11
rs4665972	C	T	0.607	-0.024	0.002	2.89E-22
rs11373337	NA	A	0.705	0.015	0.003	6.74E-09
rs6440008	C	T	0.386	-0.017	0.002	4.07E-12
rs12496573	A	G	0.211	0.016	0.003	3.36E-08
rs2035562	G	A	0.672	-0.014	0.003	4.02E-08
rs1229984	C	T	0.978	-0.056	0.008	3.82E-12
rs9291156	G	A	0.455	-0.014	0.002	7.09E-09
rs2057655	A	G	0.185	-0.017	0.003	2.31E-08
rs13188076	T	G	0.232	-0.020	0.003	2.22E-12
rs56108664	T	C	0.199	0.020	0.003	4.29E-11
rs34867029	T	NA	0.704	-0.015	0.003	3.94E-08
rs2455357	G	A	0.705	0.016	0.003	4.25E-08
rs6452788	A	G	0.236	-0.021	0.003	1.79E-13
rs2394893	A	G	0.605	0.014	0.002	2.27E-08
rs791903	C	G	0.556	0.013	0.002	2.72E-08
rs62458486	A	G	0.226	-0.016	0.003	2.37E-08
rs372106593	NA	G	0.446	-0.013	0.002	4.30E-08
rs11514731	G	C	0.179	-0.020	0.003	1.28E-10
rs12700027	A	G	0.110	0.023	0.004	2.07E-09
rs33951980	T	C	0.130	-0.021	0.004	7.08E-09
rs4873492	T	C	0.170	0.020	0.003	6.29E-10
rs11103388	A	G	0.330	0.014	0.003	2.75E-08
**UNa/UCr**						
**SNP**	**EA**	**OA**	**EAF**	**BETA**	**SE**	**P**
rs12363886	A	C	0.482	-0.013	0.002	3.29E-08

rs11062590	G	C	0.092	-0.025	0.004	2.42E-09
rs35335867	NA	C	0.248	-0.019	0.003	1.15E-11
rs11659764	A	T	0.054	0.030	0.005	3.08E-08
rs8096658	G	C	0.488	-0.014	0.002	7.96E-09
rs1698114	G	A	0.484	0.015	0.003	9.36E-09
rs1047891	A	C	0.316	-0.027	0.003	1.34E-24
rs35169442	C	G	0.198	0.021	0.003	9.87E-12
rs1260326	C	T	0.607	-0.028	0.002	4.05E-29
rs62140396	A	G	0.118	0.022	0.004	5.33E-09
rs13056137	A	C	0.264	0.017	0.003	2.53E-10
rs6440008	C	T	0.386	-0.017	0.003	4.65E-12
rs2035561	C	T	0.671	-0.014	0.003	3.29E-08
rs1229984	C	T	0.978	-0.050	0.008	7.36E-10
rs13127170	G	A	0.552	0.014	0.002	1.61E-08
rs4697700	C	G	0.236	0.016	0.003	1.17E-08
rs13188076	T	G	0.232	-0.024	0.003	6.48E-17
rs1423553	C	T	0.672	0.014	0.003	3.75E-08
rs702634	A	G	0.692	0.016	0.003	6.62E-10
rs13242739	A	T	0.051	-0.031	0.006	2.37E-08
rs33951980	T	C	0.130	-0.023	0.004	1.44E-10
rs2953661	C	A	0.696	-0.017	0.003	4.02E-09
rs2954021	G	A	0.506	-0.016	0.002	1.36E-10
rs558455	G	A	0.580	0.016	0.002	1.67E-10
rs4873492	T	C	0.170	0.022	0.003	1.07E-11
rs12378270	A	G	0.061	0.032	0.006	6.49E-09
**UK/UCr**						
**SNP**	**EA**	**OA**	**EAF**	**BETA**	**SE**	**P**
rs35041900	T	C	0.093	-0.024	0.004	1.74E-09
rs66495454	NA	G	0.378	0.014	0.002	9.63E-09
rs2664284	C	G	0.255	0.018	0.003	5.84E-12
rs4408310	A	C	0.539	0.014	0.002	4.33E-09
rs57739486	T	C	0.158	0.017	0.003	2.75E-08
rs35335867	NA	C	0.248	-0.024	0.003	8.42E-19
rs2472297	T	C	0.267	0.021	0.003	4.44E-16
rs11648252	G	C	0.795	0.016	0.003	2.09E-08
rs71352040	A	T	0.071	0.025	0.004	1.17E-08
rs4803380	T	C	0.022	-0.047	0.008	1.53E-09
rs34783010	T	G	0.193	0.026	0.003	3.10E-19
rs2603274	C	A	0.817	0.018	0.003	1.53E-09
rs1047891	A	C	0.316	-0.031	0.002	1.60E-37

rs4973766	T	C	0.456	-0.022	0.002	4.29E-21
rs1664781	A	G	0.693	0.014	0.002	1.75E-08
rs72704785	A	G	0.179	-0.024	0.003	1.03E-14
rs10872423	T	C	0.194	-0.019	0.003	2.46E-11
rs3129996	C	A	0.906	-0.021	0.004	4.20E-08
rs2260051	T	A	0.558	-0.013	0.002	1.44E-08
rs9269041	A	G	0.647	-0.014	0.002	1.89E-09
rs77213792	G	C	0.068	-0.027	0.005	2.78E-09
rs10950655	C	T	0.505	0.013	0.002	4.53E-09
rs756018	G	C	0.110	-0.026	0.004	1.19E-12
**UAlb/UCr**						
**SNP**	**EA**	**OA**	**EAF**	**BETA**	**SE**	**P**
rs35388680	NA	C	0.407	0.013	0.002	1.92E-08
rs35202981	G	A	0.140	0.020	0.003	3.03E-09
rs58953262	NA	C	0.521	0.013	0.002	4.48E-08
rs1204672	C	T	0.572	0.014	0.002	5.35E-09
rs12032996	A	G	0.162	-0.018	0.003	1.59E-08
rs17358122	T	C	0.220	-0.015	0.003	3.71E-08
rs55668525	T	G	0.192	0.017	0.003	4.03E-09
rs10157710	T	C	0.801	0.025	0.003	4.48E-18
rs3781470	A	C	0.355	0.013	0.002	3.11E-08
rs79051478	C	G	0.104	0.058	0.004	1.50E-52
rs780836	C	G	0.715	0.014	0.003	2.56E-08
rs188126412	T	C	0.028	0.073	0.007	6.58E-24
rs116867125	A	G	0.019	0.059	0.009	6.08E-12
rs79386054	T	C	0.017	0.055	0.010	9.79E-09
rs7910002	C	G	0.698	0.014	0.003	2.45E-08
rs67339103	A	G	0.212	0.020	0.003	7.90E-12
rs7115200	G	T	0.440	0.013	0.002	1.14E-08
rs34183365	NA	C	0.415	0.014	0.002	3.73E-08
rs2601006	T	C	0.343	-0.014	0.002	2.14E-08
rs8036643	C	G	0.645	0.015	0.002	8.72E-10
rs35335867	NA	C	0.248	-0.019	0.003	1.33E-12
rs146311723	C	T	0.174	0.017	0.003	2.19E-08
rs2472297	T	C	0.267	0.025	0.003	2.65E-21
rs9673084	A	G	0.271	0.019	0.003	3.06E-13
rs4488444	G	A	0.756	-0.016	0.003	7.55E-09
rs11078597	C	T	0.186	0.017	0.003	1.87E-08
rs35572189	A	G	0.361	-0.013	0.002	3.82E-08
rs784257	C	T	0.813	-0.017	0.003	5.10E-09

rs11882796	T	A	0.542	0.014	0.002	3.58E-09
rs10419198	T	C	0.250	-0.015	0.003	4.20E-08
rs10207567	C	G	0.812	0.020	0.003	3.51E-11
rs1047891	A	C	0.316	-0.018	0.002	8.39E-13
rs35687319	T	C	0.128	0.022	0.004	4.95E-10
rs4665972	C	T	0.607	-0.017	0.002	4.31E-13
rs2627770	T	C	0.732	-0.015	0.003	1.01E-08
rs12714144	T	A	0.127	-0.021	0.003	1.91E-09
rs2295762	G	A	0.360	0.014	0.002	2.11E-08
rs112607182	T	C	0.075	0.029	0.005	3.55E-10
rs6535594	A	G	0.495	0.013	0.002	1.17E-08
rs4109437	A	G	0.038	0.057	0.006	2.58E-21
rs146996008	NA	A	0.419	0.014	0.002	5.57E-09
rs13179493	C	T	0.293	-0.014	0.003	3.09E-08
rs114118094	T	A	0.075	-0.030	0.004	8.96E-12
rs40480	G	C	0.362	0.014	0.002	2.75E-09
rs10947788	T	C	0.289	0.015	0.003	1.41E-08
rs4410790	C	T	0.634	0.024	0.002	1.00E-22
rs3735533	C	T	0.926	0.025	0.004	2.36E-08
rs17158386	A	G	0.261	0.019	0.003	2.45E-12
rs28601761	G	C	0.420	-0.017	0.002	4.03E-13

Abbreviations: UNa/UK, urinary sodium to potassium ratio; UNa/UCr, urinary sodium to creatinine ratio; UK/UCr, urinary potassium to creatinine ratio; UAlb/UCr, urinary albumin to creatinine ratio; SNP, single-nucleotide polymorphism; EA, effect allele; OA, other allele; EAF, effect allele frequency; SE, standard error; P, p-value.

Table S2. Description of data used and statistical power for Mendelian randomization.

			UNa/UK				UNa/UCr				UK/UCr				UAlb/UCr			
Outcomes	PMID	Sample size	R2*	Effect obs	Power obs effect	Power fixed effect	R2*	Effect obs	Power obs effect	Power fixed effect	R2*	Effect obs	Power obs effect	Power fixed effect	R2*	Effect obs	Power obs effect	Power fixed effect
**AF**	**30061737**	1,030,836	0.0031	0.898	30	46	0.0031	0.905	26	46	0.0031	0.986	4	46	0.0063	1.155	**78**	**76**
**CAD**	**26343387**	184,305	0.0032	0.962	7	36	0.0032	0.936	11	36	0.0028	0.947	8	32	0.0060	1.042	9	59
**Stroke**	**29531354**	446,696	0.0031	0.929	12	32	0.0033	0.879	30	34	0.0030	0.892	22	31	0.0063	1.059	14	57
**T2D**	**30054458**	659,316	0.0018	0.968	5	30	0.0025	0.990	3	40	0.0018	1.036	6	30	0.0034	1.243	**88**	51
**SBP**		337,222	0.0034	0.124	**99**	**100**	0.0033	0.120	**98**	**100**	0.0031	-0.009	5	**100**	0.0064	0.157	**100**	**100**
**DBP**		337,222	0.0034	0.076	73	**100**	0.0033	0.078	74	**100**	0.0031	0.001	3	**100**	0.0064	0.136	**100**	**100**
**SBP**	**28739976**	150,134	0.0028	0.124	72	**87**	0.0029	0.120	70	**88**	0.0027	-0.009	4	**86**	0.0055	0.157	**100**	**99**
**SBP**	**28739976**	150,134	0.0028	0.076	35	**87**	0.0029	0.078	37	**88**	0.0027	0.001	3	**86**	0.0055	0.136	**98**	**99**
**BMI**	**25673413**	339,224	0.0022	0.049	26	**98**	0.0021	-0.054	31	**98**	0.0019	-0.154	**98**	**98**	0.0039	-0.170	**100**	**100**
**WHR**	**25673412**	210,082	0.0022	0.044	15	**90**	0.0021	-0.007	4	**88**	0.0019	-0.076	34	**85**	0.0039	-0.062	42	**99**

Characteristics of the consortia used in our study: number of samples, proportion of phenotypic variance explained by the instruments (tested in UK Biobank), statistical power considering the effect from observational analyses and for a fixed effect of 1.15 (binary traits) and 0.15 (continuous traits). Power estimates >75% in bold.

Abbreviations: effect obs, effect from observational analyses; AF, atrial fibrillation; CAD, coronary artery disease; T2D, type 2 diabetes; SBP and DBP, systolic and diastolic blood pressure, respectively; BMI, body mass index; WHR, waist-to-hip ratio.

* The variance explained varied somewhat across different outcomes due to lack of variants in some GWAS summary statistics.

Table S3. Observational associations of the urinary sodium to potassium ratio (UNa/UK), sodium and potassium adjusted for creatinine (UNa/UCr and UK/UCr), and urinary albumin to creatinine ratio (UAlb/UCr) with cardiovascular outcomes in UK Biobank using multivariable-adjusted Cox proportional hazards models, and multivariable-adjusted linear and logistic regression for main and sensitivity analyses.

UNa/UK	Main					Sensitivity				
**Outcome**	**N**	**HR/OR/Beta**	**Lower**	**Upper**	**P**	**N**	**HR/OR/Beta**	**Lower**	**Upper**	**P**
**AF**	9,196	0.898	0.879	0.917	**<2e-16**	5,499	0.895	0.869	0.921	**5.03E-14**
**CAD**	7,375	0.962	0.939	0.985	**1.24E-03**	4,659	0.977	0.947	1.009	1.53E-01
**HF**	2,775	0.960	0.924	0.997	3.41E-02	1,422	0.999	0.944	1.057	9.72E-01
**HS**	978	0.982	0.92	1.049	5.91E-01	714	0.967	0.893	1.047	4.07E-01
**IS**	1,888	0.929	0.886	0.973	**1.94E-03**	1,268	0.946	0.891	1.004	6.69E-02
**LIPID**	67,898	0.940	0.93	0.957	**<2e-16**	28,978	0.913	0.899	0.926	**<2e-16**
**T2D**	19,876	0.968	0.951	0.984	**1.84E-04**	7,758	0.986	0.959	1.014	3.16E-01
**SBP**		0.124	0.121	0.127	**<2e-16**		0.131	0.127	0.134	**<2e-16**
**DBP**		0.076	0.073	0.079	**<2e-16**		0.080	0.077	0.083	**<2e-16**
**BF**		0.026	0.024	0.029	**<2e-16**		0.022	0.019	0.024	**<2e-16**
**BMI**		0.049	0.047	0.052	**<2e-16**		0.049	0.046	0.052	**<2e-16**
**WHR**		0.044	0.042	0.047	**<2e-16**		0.045	0.042	0.048	**<2e-16**
**UNa/UCr**	**Main**					**Sensitivity**				
**Outcome**	**N**	**HR/OR/Beta**	**Lower**	**Upper**	**P**	**N**	**HR/OR/Beta**	**Lower**	**Upper**	**P**
**AF**	9,219	0.905	0.887	0.924	**<2e-16**	5,516	0.895	0.87	0.922	**8.48E-14**
**CAD**	7,387	0.936	0.914	0.958	**1.93E-08**	4,665	0.942	0.913	0.973	**2.37E-04**
**HF**	2,776	0.937	0.904	0.972	**4.76E-04**	1,423	0.967	0.914	1.024	2.47E-01
**HS**	978	0.955	0.897	1.018	1.60E-01	714	0.965	0.892	1.045	3.82E-01
**IS**	1,888	0.879	0.84	0.92	**3.04E-08**	1,268	0.881	0.83	0.935	**3.26E-05**

**LIPID**	67,898	0.958	0.948	0.967	**<2e-16**	28,978	0.942	0.928	0.956	**2.46E-15**
**T2D**	19,876	0.990	0.974	1.006	2.25E-01	7,758	1.024	0.995	1.053	1.02E-01
**SBP**		0.120	0.118	0.123	**<2e-16**		0.128	0.125	0.131	**<2e-16**
**DBP**		0.078	0.076	0.081	**<2e-16**		0.084	0.08	0.087	**<2e-16**
**BF**		-0.040	-0.042	-0.037	**<2e-16**		-0.048	-0.05	-0.045	**<2e-16**
**BMI**		-0.054	-0.057	-0.051	**<2e-16**		-0.061	-0.064	-0.058	**<2e-16**
**WHR**		-0.007	-0.009	-0.005	**1.58E-09**		-0.009	-0.011	-0.006	**2.08E-11**
**UK/UCr**	**Main**					**Sensitivity**				
**Outcome**	**N**	**HR/OR/Beta**	**Lower**	**Upper**	**P**	**N**	**HR/OR/Beta**	**Lower**	**Upper**	**P**
**AF**	9,229	0.986	0.962	1.009	2.35E-01	5,513	0.997	0.967	1.028	8.50E-01
**CAD**	7,385	0.947	0.922	0.972	**5.46E-05**	4,666	0.945	0.914	0.977	**9.22E-04**
**HF**	2,789	0.916	0.877	0.956	**6.07E-05**	1,425	0.934	0.879	0.992	2.52E-02
**HS**	979	0.942	0.879	1.011	9.83E-02	715	0.984	0.907	1.067	6.95E-01
**IS**	1,894	0.892	0.847	0.939	**1.57E-05**	1,270	0.890	0.836	0.948	**2.89E-04**
**LIPID**	67,898	1.030	1.018	1.043	**6.52E-07**	28,978	1.050	1.034	1.067	**8.47E-10**
**T2D**	19,876	1.036	1.016	1.057	**4.82E-04**	7,758	1.051	1.02	1.083	**1.10E-03**
**SBP**		-0.009	-0.012	-0.006	**2.80E-09**		-0.015	-0.018	-0.012	**<2e-16**
**DBP**		0.001	-0.002	0.004	5.45E-01		-0.003	-0.007	0	4.56E-02
**BF**		-0.099	-0.101	-0.096	**<2e-16**		-0.095	-0.098	-0.093	**<2e-16**
**BMI**		-0.154	-0.157	-0.151	**<2e-16**		-0.150	-0.153	-0.147	**<2e-16**
**WHR**		-0.076	-0.079	-0.074	**<2e-16**		-0.074	-0.077	-0.071	**<2e-16**
**UAlb/UCr**	**Main**					**Sensitivity**				
**Outcome**	**N**	**HR/OR/Beta**	**Lower**	**Upper**	**P**	**N**	**HR/OR/Beta**	**Lower**	**Upper**	**P**
**AF**	9,251	1.155	1.13	1.18	**<2e-16**	5,529	1.113	1.081	1.146	**4.40E-13**
**CAD**	7,397	1.042	1.017	1.067	**6.87E-04**	4,672	1.015	0.984	1.047	3.56E-01
**HF**	2,791	1.226	1.181	1.272	**<2e-16**	1,425	1.210	1.145	1.28	**1.61E-11**

**HS**	979	1.153	1.077	1.233	**3.90E-05**	715	1.132	1.043	1.228	**2.95E-03**
**IS**	1,895	1.059	1.01	1.111	1.82E-02	1,271	1.031	0.971	1.095	3.22E-01
**LIPID**	67,898	1.056	1.045	1.068	**<2e-16**	28,978	1.060	1.044	1.076	**2.61E-14**
**T2D**	19,876	1.243	1.222	1.265	**<2e-16**	7,758	1.226	1.193	1.26	**<2e-16**
**SBP**		0.157	0.154	0.16	**<2e-16**		0.161	0.157	0.164	**<2e-16**
**DBP**		0.136	0.133	0.139	**<2e-16**		0.145	0.141	0.148	**<2e-16**
**BF**		-0.106	-0.108	-0.103	**<2e-16**		-0.114	-0.117	-0.112	**<2e-16**
**BMI**		-0.170	-0.173	-0.167	**<2e-16**		-0.185	-0.189	-0.182	**<2e-16**
**WHR**		-0.062	-0.064	-0.059	**<2e-16**		-0.071	-0.074	-0.068	**<2e-16**

Main analyses were performed excluding participants with kidney disease and/or cardiovascular disease at baseline. Sensitivity analyses was performed excluding participants with kidney disease and/or cardiovascular disease at baseline and/or participants using medications affecting renal excretion. Estimates are from multivariable-adjusted linear (continuous risk factors), Cox proportional hazards models (cardiovascular outcomes) or logistic (lipid treatment and T2D) regression. The estimates represent SD change in outcome variable per SD change in the urinary biomarker tested. Binary outcomes: atrial fibrillation (AF), coronary artery disease (CAD), heart failure (HF), hemorrhagic stroke (HS), ischemic stroke (IS), lipid medications (LIP) and type 2 diabetes (T2D). Continuous outcomes: systolic and diastolic blood pressure (SBP, and DBP; respectively), body fat percentage (BF), body mass index (BMI), waist-to-hip ratio (WHR). Models are adjusted for sex, age, center in UK, ethnicity, smoking, blood pressure (SBP and DBP), medication, lipids, T2D, obesity (BMI, WHR, body fat percentage) alcohol, Townsend index. P-values significant after Bonferroni correction ($p \leq 4.17E-03$) are bold. N, incident events in the UK Biobank. Abbreviations: HR, hazard ratio; OR, odds ratio; CI, confidence interval; P, p-value.

Table S4. Observational associations of the urinary sodium to potassium ratio (UNa/UK) with cardiovascular outcomes in UK Biobank using multivariable-adjusted Cox proportional hazards models, and multivariable-adjusted linear and logistic regression for main and sensitivity analyses. Models are adjusted for **a.** sex, age, center in UK, ethnicity, smoking, medication, lipids, T2D, obesity (BMI, WHR, body fat percentage) alcohol, and Townsend index; **b.** sex, age, center in UK, and ethnicity.

a.

**UNa/UK**	**Main**					**Sensitivity**				
**Outcome**	**N**	**HR/OR/Beta**	**Lower**	**Upper**	**P**	**N**	**HR/OR/Beta**	**Lower**	**Upper**	**P**
**AF**	9,205	0.891	0.873	0.91	**<2e-16**	5501	0.892	0.867	0.918	**6.40E-15**
**CAD**	7,378	0.986	0.963	1.01	2.53E-01	4660	1.013	0.982	1.046	4.03E-01
**HF**	2,777	0.959	0.924	0.996	3.01E-02	1424	1.024	0.968	1.083	4.06E-01
**HS**	979	0.997	0.935	1.064	9.31E-01	715	0.992	0.917	1.073	8.41E-01
**IS**	1,892	0.958	0.915	1.003	6.98E-02	1271	0.98	0.924	1.04	5.15E-01
**LIPID**		0.933	0.923	0.944	**<2e-16**		0.910	0.895	0.925	**<2e-16**
**T2D**		0.960	0.943	0.977	**2.67E-06**		0.985	0.957	1.012	2.68E-01
**BF**		0.043	0.041	0.045	**<2e-16**		0.043	0.040	0.045	**<2e-16**
**BMI**		0.075	0.072	0.078	**<2e-16**		0.081	0.078	0.084	**<2e-16**
**WHR**		0.056	0.054	0.059	**<2e-16**		0.060	0.058	0.063	**<2e-16**

b.

**UNa/UK**	**Main**					**Sensitivity**				
**Outcome**	**N**	**HR/OR/B**	**Lower**	**Upper**	**P**	**N of events**	**HR/OR/B**	**Lower**	**Upper**	**P**
**AF**	10356	0.936	0.918	0.955	**1.02E-10**	6080	0.916	0.892	0.941	**2.35E-10**
**CAD**	8130	1.045	1.022	1.069	**1.39E-04**	5076	1.063	1.032	1.095	**5.55E-05**
**HF**	3328	1.049	1.012	1.086	8.14E-03	1639	1.077	1.023	1.135	4.99E-03
**HS**	1071	0.998	0.939	1.062	9.57E-01	766	1.004	0.931	1.082	9.18E-01
**IS**	2127	1.012	0.969	1.057	5.92E-01	1397	1.036	0.979	1.095	2.23E-01
**LIPID**		1.044	1.035	1.052	**<2e-16**		0.956	0.943	0.969	**3.94E-11**
**T2D**		1.101	1.085	1.116	**<2e-16**		1.058	1.033	1.082	**8.03E-06**
**SBP**		0.137	0.134	0.139	**<2e-16**		0.138	0.135	0.141	**<2e-16**
**DBP**		0.095	0.092	0.097	**<2e-16**		0.094	0.091	0.097	**<2e-16**
**BF**		0.051	0.048	0.053	**<2e-16**		0.040	0.038	0.043	**<2e-16**
**BMI**		0.089	0.086	0.092	**<2e-16**		0.080	0.077	0.084	**<2e-16**
**WHR**		0.066	0.064	0.069	**<2e-16**		0.061	0.059	0.064	**<2e-16**

Main analyses were performed excluding participants with kidney disease and/or cardiovascular disease at baseline. Sensitivity analyses was performed excluding participants with kidney disease and/or cardiovascular disease at baseline and/or participants using medications affecting renal excretion.
Estimates are from multivariable-adjusted linear (continuous risk factors), Cox proportional hazards models (cardiovascular outcomes) or logistic (lipid treatment and T2D) regression. The estimates represent SD change in outcome variable per SD change in the urinary biomarker tested. Binary outcomes: atrial fibrillation (AF), coronary artery disease (CAD), heart failure (HF), hemorrhagic stroke (HS), ischemic stroke (IS), lipid medications (LIP) and type 2 diabetes (T2D).
Continuous outcomes: body fat percentage (BF), body mass index (BMI), waist-to-hip ratio (WHR). P-values significant after Bonferroni correction ($p \leq 4.17E-03$) are bold. N, incident events in the UK Biobank.
Abbreviations: HR, hazard ratio; OR, odds ratio; CI, confidence interval; P, p-value.

Table S5. Observational associations of urinary sodium, potassium and albumin with cardiovascular risk factors in UK Biobank using multivariable-adjusted logistic regression for main analyses.

Sodium	Beta	lower	upper	P
**BF**	0.0022	0.0022	0.0023	**<2e-16**
**BMI**	0.0038	0.0037	0.0039	**<2e-16**
**WHR**	0.0022	0.0021	0.0022	**<2e-16**
**Potassium**				
**BF**	0.0021	0.0020	0.0021	**<2e-16**
**BMI**	0.0034	0.0033	0.0034	**<2e-16**
**WHR**	0.0015	0.0014	0.0015	**<2e-16**
**Albumin**				
**BF**	0.0001	0.0001	0.0002	**1.46E-09**
**BMI**	0.0003	0.0002	0.0003	**<2e-16**
**WHR**	0.0003	0.0002	0.0003	**<2e-16**

Continuous outcomes: body fat percentage (BF), body mass index (BMI), waist-to-hip ratio (WHR). Models are adjusted for sex, age, center in UK, ethnicity, smoking, blood pressure, medication, lipids, type two diabetes, obesity (BMI, WHR, body fat percentage) alcohol, Townsend index. Significant p-values in bold.
Abbreviations: P, p-value.

Table S6. Sex-stratified associations of the urinary sodium to potassium ratio (UNa/UK), sodium and potassium adjusted for creatinine (UNa/UCr and UK/UCr), and urinary albumin to creatinine ratio (UAlb/UCr) with cardiovascular outcomes in UK Biobank using multivariable-adjusted Cox proportional hazards models, and multivariable-adjusted linear and logistic regression for main analysis.

**UNa/UK**											
**Females**						**Males**					
**Outcome**	**N**	**HR/OR/Beta**	**Lower**	**Higher**	**P**	**N**	**HR/OR/Beta**	**Lower**	**Higher**	**P**	**P interaction**
**AF**	3,329	0.917	0.886	0.949	**6.22E-07**	5,867	0.887	0.863	0.911	**<2e-16**	4.25E-02
**CAD**	1,925	0.992	0.948	1.037	7.15E-01	5,450	0.949	0.923	0.976	**2.78E-04**	7.59E-03
**HF**	971	0.979	0.92	1.042	5.13E-01	1,804	0.946	0.902	0.993	2.30E-02	1.90E-01
**HS**	487	1.02	0.931	1.117	6.75E-01	491	0.94	0.857	1.033	1.98E-01	9.09E-02
**IS**	720	0.963	0.894	1.037	3.18E-01	1,168	0.904	0.851	0.96	**1.00E-03**	1.70E-01
**LIPIDS**	28,711	0.945	0.931	0.959	**3.25E-13**	39,186	0.937	0.923	0.950	**<2e-16**	1.24E-06
**T2D**	8,375	0.999	0.973	1.025	9.37E-01	11,501	0.939	0.917	0.961	**8.73E-08**	1.01E-03
**SBP**		0.131	0.127	0.135	**<2e-16**		0.116	0.112	0.120	**<2e-16**	9.14E-01
**DBP**		0.072	0.068	0.076	**<2e-16**		0.081	0.077	0.086	**<2e-16**	7.79E-05
**BF**		0.017	0.014	0.020	**<2e-16**		0.038	0.035	0.041	**<2e-16**	2.00E-03
**BMI**		0.043	0.039	0.047	**<2e-16**		0.058	0.054	0.062	**<2e-16**	1.11E-01
**WHR**		0.035	0.032	0.038	**<2e-16**		0.056	0.053	0.059	**<2e-16**	2.01E-04
**UNa/UCr**											
**Females**						**Males**					
**Outcome**	**N**	**HR/OR/Beta**	**Lower**	**Higher**	**P**	**N**	**HR/OR/Beta**	**Lower**	**Higher**	**P**	**P interaction**
**AF**	3,341	0.914	0.886	0.943	**2.30E-08**	5,878	0.898	0.875	0.923	**5.88E-15**	1.83E-01
**CAD**	1,929	0.941	0.903	0.981	5.00E-03	5,458	0.933	0.907	0.96	**1.30E-06**	2.59E-01
**HF**	972	0.954	0.901	1.01	1.06E-01	1,804	0.925	0.882	0.969	**1.00E-03**	2.04E-01

**HS**	487	1.014	0.93	1.106	7.45E-01	491	0.888	0.808	0.975	1.30E-02	1.59E-02
**IS**	719	0.895	0.835	0.959	**2.00E-03**	1,169	0.865	0.814	0.919	**2.68E-06**	4.87E-01
**LIPIDS**	28,711	0.958	0.945	0.972	**3.39E-09**	39,186	0.960	0.946	0.974	**3.97E-08**	8.61E-01
**T2D**	8,375	0.986	0.962	1.010	2.56E-01	11,501	0.991	0.969	1.014	4.59E-01	3.17E-01
**SBP**		0.120	0.117	0.124	**<2e-16**		0.119	0.115	0.123	**<2e-16**	6.89E-01
**DBP**		0.073	0.069	0.076	**<2e-16**		0.087	0.082	0.091	**<2e-16**	6.08E-05
**BF**		-0.050	-0.053	-0.047	**<2e-16**		-0.023	-0.026	-0.020	**<2e-16**	<2e-16
**BMI**		-0.066	-0.070	-0.063	**<2e-16**		-0.034	-0.037	-0.030	**<2e-16**	<2e-16
**WHR**		-0.012	-0.015	-0.009	**5.21E-16**		0.002	-0.001	0.005	2.52E-01	1.66E-05
**UK/UCr**											
**Females**						**Males**					
**Outcome**	**N**	**HR/OR/Beta**	**Lower**	**Higher**	**P**	**N**	**HR/OR/Beta**	**Lower**	**Higher**	**P**	**P interaction**
**AF**	3,349	0.969	0.934	1.006	9.70E-02	5,880	0.998	0.967	1.029	8.79E-01	2.20E-01
**CAD**	1,932	0.912	0.869	0.958	**2.23E-04**	5,453	0.964	0.934	0.995	2.50E-02	2.09E-02
**HF**	978	0.913	0.852	0.978	9.00E-03	1,811	0.919	0.869	0.971	**3.00E-03**	8.76E-01
**HS**	488	0.983	0.894	1.081	7.18E-01	491	0.901	0.811	1	5.00E-02	3.30E-01
**IS**	725	0.867	0.801	0.939	**4.14E-04**	1,169	0.911	0.851	0.977	8.00E-03	2.76E-01
**LIPIDS**	28,711	1.018	1.001	1.035	3.71E-02	39,186	1.046	1.029	1.064	**8.38E-08**	3.19E-11
**T2D**	8,375	0.971	0.943	1.000	5.39E-02	11,501	1.103	1.073	1.134	**2.06E-12**	3.89E-08
**SBP**		-0.014	-0.018	-0.010	**1.78E-12**		-0.006	-0.010	-0.001	9.71E-03	3.28E-05
**DBP**		0.001	-0.003	0.005	6.32E-01		-0.0005	-0.005	0.004	8.42E-01	1.57E-02
**BF**		-0.099	-0.102	-0.096	**<2e-16**		-0.095	-0.099	-0.092	**<2e-16**	6.76E-16
**BMI**		-0.160	-0.164	-0.156	**<2e-16**		-0.141	-0.145	-0.137	**<2e-16**	<2e-16
**WHR**		-0.068	-0.071	-0.065	**<2e-16**		-0.086	-0.090	-0.083	**<2e-16**	1.50E-02
**UAlb/UCr**											
**Females**						**Males**					

Outcome	N	HR/OR/Beta	Lower	Higher	P	N	HR/OR/Beta	Lower	Higher	P	P interaction
**AF**	3,362	1.125	1.08	1.172	**1.28E-08**	5,889	1.176	1.147	1.206	**<2e-16**	4.39E-01
**CAD**	1,938	1.066	1.011	1.123	1.80E-02	5,459	1.049	1.021	1.077	**4.84E-04**	1.90E-02
**HF**	980	1.243	1.156	1.336	**4.40E-09**	1,811	1.228	1.175	1.283	**<2e-16**	2.23E-01
**HS**	488	1.083	0.974	1.204	1.42E-01	491	1.201	1.1	1.312	**4.61E-05**	3.44E-01
**IS**	725	1.027	0.942	1.12	5.44E-01	1,170	1.077	1.017	1.141	1.10E-02	5.05E-01
**LIPIDS**	28,711	1.056	1.037	1.075	**4.14E-09**	39,186	1.065	1.050	1.080	**<2e-16**	7.93E-15
**T2D**	8,375	1.116	1.083	1.150	**<2e-16**	11,501	1.316	1.288	1.345	**<2e-16**	<2e-16
**SBP**		0.171	0.167	0.175	**<2e-16**		0.150	0.147	0.154	**<2e-16**	<2e-16
**DBP**		0.139	0.134	0.143	**<2e-16**		0.137	0.133	0.142	**<2e-16**	6.95E-04
**BF**		-0.152	-0.156	-0.149	**<2e-16**		-0.059	-0.062	-0.056	**<2e-16**	<2e-16
**BMI**		-0.232	-0.237	-0.228	**<2e-16**		-0.099	-0.103	-0.095	**<2e-16**	<2e-16
**WHR**		-0.087	-0.091	-0.084	**<2e-16**		-0.033	-0.036	-0.030	**<2e-16**	<2e-16

Sex-stratified associations in the main cohort were performed excluding participants with kidney disease and/or cardiovascular disease at baseline. Estimates are from multivariable-adjusted linear (continuous risk factors), Cox proportional hazards models (cardiovascular outcomes) or logistic (lipid treatment and T2D) regression. The estimates represent SD change in outcome variable per SD change in urinary biomarkers. Binary outcomes: atrial fibrillation (AF), coronary artery disease (CAD), heart failure (HF), hemorrhagic stroke (HS), ischemic stroke (IS), lipid medications (LIP) and type 2 diabetes (T2D). Continuous outcomes: systolic and diastolic blood pressure (SBP, and DBP; respectively), body fat percentage (BF), body mass index (BMI), waist-to-hip ratio (WHR). Associations are adjusted for sex, age, center in UK, ethnicity, smoking, blood pressure [SBP and DBP], medication, lipids, T2D, obesity [BMI, WHR, body fat percentage] alcohol, Townsend index. P-values significant after Bonferroni correction ($p \leq 4.17E-03$) are bold. N, incident events in the UK Biobank. Abbreviations: HR, hazard ratio; OR, odds ratio; CI, confidence interval; P, p-value.

Table S7. Mendelian randomization analyses of associations of the urinary sodium to potassium ratio (UNa/UK), sodium and potassium adjusted for creatinine (UNa/UCr and UK/UCr) and urinary albumin to creatinine ratio (UAlb/UCr) with different cardiovascular risk factors and outcomes.

UNa/UK								
**Method**	**N SNP**	**Beta**	**SE**	**P**	**Global test P**	**Outlier corrected Beta**	**SD**	**P**
**SBP UKB**					<0.001	n outliers = 8		
**IVW**	26	2.118	1.876	2.59E-01		2.630	1.093	**2.59E-02**
**MR Egger**	26	-9.190	8.238	2.76E-01				
**WM**	26	0.941	1.267	4.58E-01				
**SBP ICBP**					<0.001	n outliers = 3		
**IVW**	20	1.981	1.667	2.35E-01		0.496	1.214	6.87E-01
**MR Egger**	20	-4.085	6.521	5.39E-01				
**WM**	20	-0.044	1.541	9.77E-01				
**DBP UKB**					<0.001	n outliers = 7		
**IVW**	26	1.243	0.844	1.41E-01		2.053	0.610	**2.81E-03**
**MR Egger**	26	1.121	3.856	7.74E-01				
**WM**	26	0.828	0.626	1.86E-01				
**DBP ICBP**					0.007	n outliers = 1		
**IVW**	20	1.194	0.832	1.51E-01		0.591	0.713	4.16E-01
**MR Egger**	20	0.831	3.356	8.07E-01				
**WM**	20	1.424	0.921	1.22E-01				
**BMI**					<0.001	n outliers = 6		
**IVW**	17	0.159	0.182	3.82E-01		0.351	0.119	**1.31E-02**
**MR Egger**	17	-0.033	1.056	9.76E-01				
**WM**	17	0.246	0.136	6.95E-02				

**WHR**					0.039	n outliers = 0		
**IVW**	17	0.220	0.086	1.05E-02				
**MR Egger**	17	0.736	0.480	1.46E-01				
**WM**	17	0.240	0.094	1.10E-02				
**UNa/UCr**								
**Method**	**N SNP**	**Beta**	**SE**	**P**	**Global test P**	**Outlier corrected Beta**	**SD**	**P**
**SBP UKB**					<0.001	n outliers = 7		
**IVW**	24	3.233	1.768	6.75E-02		3.998	1.067	**1.61E-03**
**MR Egger**	24	2.375	6.432	7.16E-01				
**WM**	24	3.265	1.159	4.85E-03				
**SBP ICBP**					<0.001	n outliers = 3		
**IVW**	20	2.660	1.551	8.62E-02		2.990	1.160	**1.90E-02**
**MR Egger**	20	3.252	5.509	5.62E-01				
**WM**	20	4.862	1.395	4.90E-04				
**DBP UKB**					<0.001	n outliers = 4		
**IVW**	24	1.663	0.716	2.01E-02		2.295	0.590	**9.12E-04**
**MR Egger**	24	3.794	2.561	1.53E-01				
**WM**	24	0.818	0.644	2.05E-01				
**DBP ICBP**					0.003	n outliers = 1		
**IVW**	20	0.956	0.769	2.14E-01		0.657	0.694	3.56E-01
**MR Egger**	20	3.472	2.670	2.10E-01				
**WM**	20	2.232	0.837	7.68E-03				
**BMI**					<0.001	n outliers = 1		
**IVW**	14	-0.289	0.098	3.20E-03		-0.368	0.072	**2.60E-04**
**MR Egger**	14	-0.286	0.432	5.20E-01				
**WM**	14	-0.389	0.081	1.60E-06				

**WHR**					0.542	n outliers = 0		
**IVW**	14	0.235	0.061	1.08E-04				
**MR Egger**	14	0.250	0.257	3.50E-01				
**WM**	14	0.217	0.086	1.14E-02				
**UK/UCr**								
**Method**	**N SNP**	**Beta**	**SE**	**P**	**Global test P**	**Outlier corrected Beta**	**SD**	**P**
**SBP UKB**					<0.001	n outliers = 2		
**IVW**	20	1.998	1.221	1.02E-01		0.694	1.140	5.50E-01
**MR Egger**	20	5.694	4.381	2.10E-01				
**WM**	20	1.790	1.163	1.24E-01				
**SBP ICBP**					<0.001	n outliers = 2		
**IVW**	17	2.195	1.998	2.72E-01		2.941	1.552	7.89E-02
**MR Egger**	17	-3.643	7.707	6.43E-01				
**WM**	17	5.035	1.613	1.80E-03				
**DBP UKB**					<0.001	n outliers = 1		
**IVW**	20	0.456	0.604	4.51E-01		-0.097	0.554	8.63E-01
**MR Egger**	20	-0.171	2.209	9.39E-01				
**WM**	20	0.532	0.618	3.89E-01				
**DBP ICBP**					<0.001	n outliers = 3		
**IVW**	17	0.700	1.357	6.06E-01		2.091	0.974	5.12E-02
**MR Egger**	17	-5.809	5.054	2.68E-01				
**WM**	17	2.127	0.985	3.08E-02				
**BMI**					<0.001	n outliers = 3		
**IVW**	11	-0.300	0.187	1.09E-01		-0.338	0.147	6.17E-02
**MR Egger**	11	-0.906	0.726	2.44E-01				
**WM**	11	-0.115	0.118	3.30E-01				

**WHR**						n outliers = 1		
**IVW**	11	0.072	0.100	4.68E-01	0.028	0.027	0.080	7.45E-01
**MR Egger**	11	-0.054	0.410	8.98E-01				
**WM**	11	0.063	0.099	5.29E-01				
**UAlb/UCr**								
**Method**	**N SNP**	**Beta**	**SE**	**P**	**Global test P**	**Outlier corrected Beta**	**SD**	**P**
**SBP UKB**					<0.001	n outliers = 5		
**IVW**	42	3.355	0.927	2.95E-04		2.753	0.671	**2.06E-04**
**MR Egger**	42	1.126	2.289	6.26E-01				
**WM**	42	2.174	0.757	4.07E-03				
**SBP ICBP**					<0.001	n outliers = 4		
**IVW**	36	2.186	1.311	9.53E-02		2.296	1.026	**3.18E-02**
**MR Egger**	36	-0.407	3.575	9.10E-01				
**WM**	36	1.372	1.151	2.34E-01				
**DBP UKB**					<0.001	n outliers = 7		
**IVW**	42	0.974	0.553	7.83E-02		0.848	0.393	**3.77E-02**
**MR Egger**	42	0.984	1.386	4.82E-01				
**WM**	42	0.967	0.455	3.34E-02				
**DBP ICBP**					<0.001	n outliers = 6		
**IVW**	36	0.602	0.854	4.80E-01		0.809	0.645	2.18E-01
**MR Egger**	36	-1.275	2.329	5.88E-01				
**WM**	36	0.260	0.695	7.08E-01				
**BMI**					<0.001	n outliers = 2		
**IVW**	29	-0.134	0.071	5.90E-02		-0.056	0.065	4.02E-01
**MR Egger**	29	0.103	0.346	7.68E-01				
**WM**	29	-0.025	0.075	7.44E-01				

**WHR**					0.178	n outliers = 0		
**IVW**	29	0.187	0.055	7.36E-04				
**MR Egger**	29	0.359	0.266	1.90E-01				
**WM**	29	0.215	0.076	4.83E-03				
**T2D**					0.003	n outliers = 1		
**IVW**	26	0.411	0.142	3.76E-03		0.282	0.117	**2.30E-02**
**MR Egger**	26	0.432	0.730	5.60E-01				
**WM**	26	0.208	0.160	1.94E-01				
**AF**					<0.001	n outliers = 4		
**IVW**	41	0.095	0.120	4.28E-01		0.105	0.086	2.32E-01
**MR Egger**	41	0.225	0.317	4.83E-01				
**WM**	41	0.058	0.113	6.09E-01				

Three separate methods were used to estimate causal effects of urinary biomarkers with different risk factors for cardiovascular disease: the standard inverse-variance weighted (IVW) regression; and two robust regression methods, the weighted median-based method (WM), and Egger regression. We applied the global test to correct for pleiotropy. Significant p-values ($p \leq 0.05$) in bold.

Abbreviations: SBP, systolic blood pressure; DBP, diastolic blood pressure; BMI, body mass index; WHR, waist-to-hip ratio; T2D, type two diabetes; AF, atrial fibrillation; N, number of SNPs; SE, standard error; SD, standard deviation; P, P-value.

Table S8. Mendelian randomization analyses of associations of systolic and diastolic blood pressure in the UK Biobank (SBP and DBP, respectively) and type two diabetes (T2D) with urinary sodium to potassium ratio (UNa/UK) and urinary albumin to creatinine ratio (UAlb/UCr).

**SBP**								
**Method**	**N SNP**	**Beta**	**SE**	**P**	**Global test P**	**Outlier corrected Beta**	**SE**	**P**
**UNa/UK**					<0.001	n outliers = 11		
**IVW**	290	0.0015	0.0007	3.42E-02		0.0018	0.0006	**2.68E-03**
**MR Egger**	290	-0.0089	0.0023	1.44E-04				
**WM**	290	0.0008	0.0008	2.75E-01				
**UAlb/UCr**					<0.001	n outliers = 12		
**IVW**	290	0.0067	0.0007	2.00E-21		0.0067	0.0006	**9.48E-24**
**MR Egger**	290	0.0063	0.0023	7.14E-03				
**WM**	290	0.0069	0.0008	1.26E-19				
**DBP**								
**Method**	**N SNP**	**Beta**	**SE**	**P**	**Global test P**	**Outlier corrected Beta**	**SE**	**P**
**UNa/UK**					<0.001	n outliers = 12		
**IVW**	253	-0.0013	0.0013	3.15E-01		-0.0006	0.0011	5.77E-01
**MR Egger**	253	-0.0140	0.0043	1.41E-03				
**WM**	253	0.0003	0.0014	8.55E-01				
**UAlb/UCr**					<0.001	n outliers = 12		
**IVW**	253	0.0084	0.0014	8.08E-10		0.0083	0.0012	**9.23E-12**
**MR Egger**	253	0.0083	0.0048	8.48E-02				
**WM**	253	0.0090	0.0014	3.37E-11				
**T2D**								
**Method**	**N SNP**	**Beta**	**SE**	**P**	**Global test P**	**Outlier corrected Beta**	**SE**	**P**

**UAlb/UCr**					<0.001	n outliers = 5		
**IVW**	156	0.0193	0.0051	1.42E-04		0.0210	0.0042	**1.89E-06**
**MR Egger**	156	0.0239	0.0126	5.98E-02				
**WM**	156	0.0253	0.0064	7.04E-05				

Three separate methods were used to estimate causal effects of urinary biomarkers with different risk factors for cardiovascular disease: the standard inverse-variance weighted (IVW) regression; and two robust regression methods, the weighted median-based method (WM), and Egger regression. We applied the global test to correct for pleiotropy. Significant p-values ($p \leq 0.05$) in bold.
Abbreviations: N, number of SNPs; SE, standard error; SD, standard deviation; P, P-value.

Table S9. Association of urinary albumin to creatinine ratio (UAlb/UCr), systolic blood pressure (SBP) and waist-to-hip ratio (WHR) with type 2 diabetes (T2D) and atrial fibrillation (AF) from mediation MR analyses to address whether UAlb/UCr had a causal effect on T2D and AF, independently of SBP and WHR.

**T2D**	**Beta**	**SE**	**P**
**UAlb/UCr**	0.4809	0.1987	**1.55E-02**
**SBP**	0.0195	0.0044	**9.06E-06**
**WHR**	0.1339	0.1018	1.88E-01
**AF**			
**UAlb/UCr**	0.0951	0.1521	5.32E-01
**SBP**	0.0124	0.0035	**3.19E-04**
**WHR**	-0.1970	0.0825	**1.70E-02**

Estimates are from multivariable MR regression-based model. Significant p-values ($p \leq 0.05$) in bold.
Abbreviations: SE, standard error; P, P-value.

Supplemental Figures

Figure S1.

a.

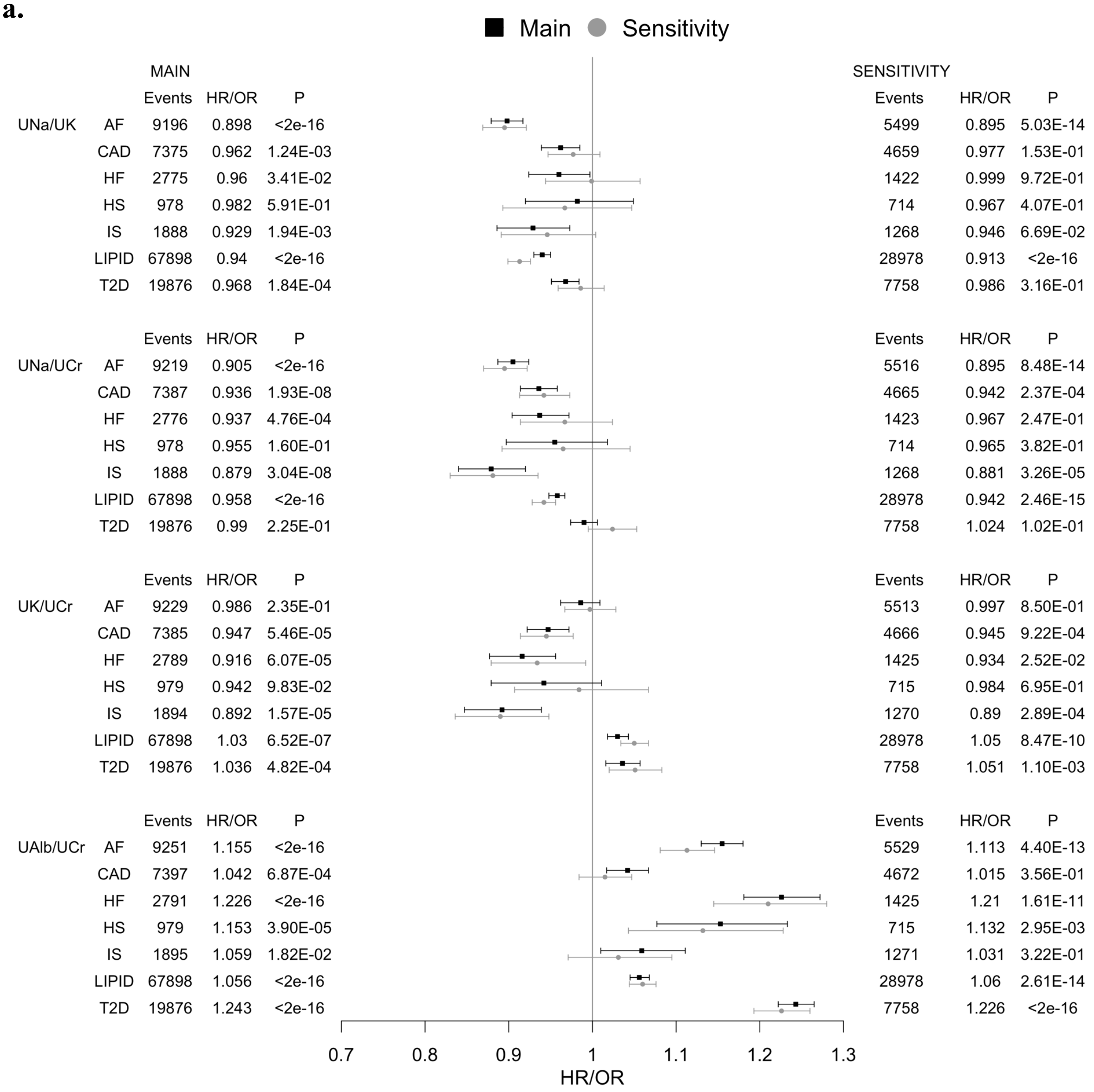

b.

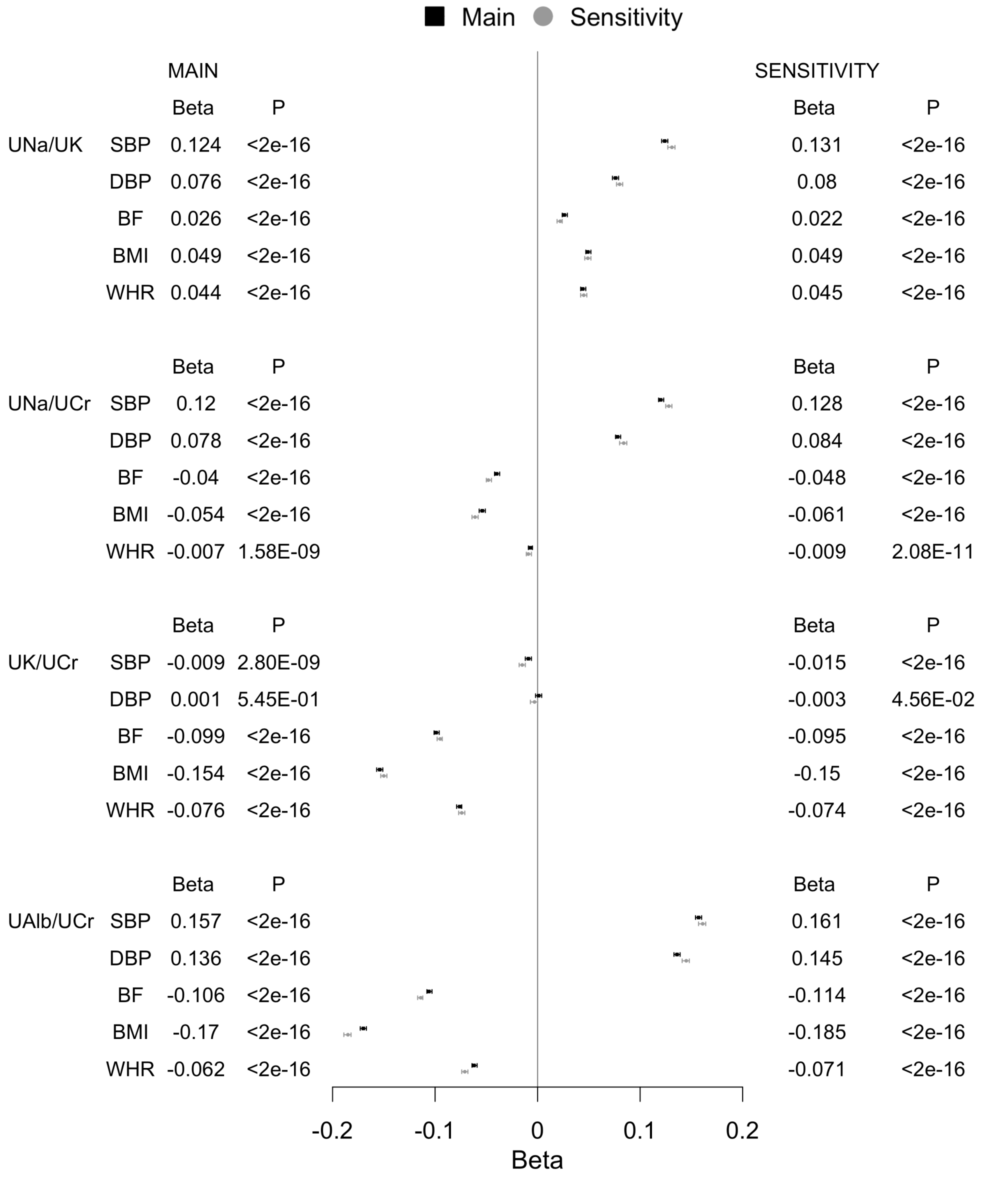

Association results (BETA/HR/OR) of urinary sodium to potassium ratio (Na/UK), urinary sodium to creatinine ratio (UNa/UCr), urinary potassium to creatinine ratio (UK/UCr) and urinary albumin to creatinine ratio (UAlb/UCr) with cardiovascular outcomes in UK Biobank using multivariable-adjusted linear and logistic regression, and multivariable-adjusted Cox proportional hazards models. a. Binary outcomes: atrial fibrillation (AF); coronary artery disease (CAD); heart failure (HF); hemorrhagic and ischemic stroke (HS and IS, respectively); lipid medications (LIP) and type 2 diabetes (T2D). **b.** Continuous outcomes:

systolic and diastolic blood pressure (SBP and DBP, respectively), body fat percentage (BF); body mass index (BMI), waist-to-hip ratio (WHR).

Main analyses were performed excluding participants with kidney disease and/or cardiovascular disease at baseline. Sensitivity analyses was performed excluding participants with kidney disease and/or cardiovascular disease at baseline and/or participants using medications affecting renal excretion. The betas from linear regression represent SD change in outcome variable per SD change in urinary biomarkers. Model adjustment: sex, age, region of the UK Biobank assessment center, ethnicity, smoking, alcohol, physical activity, Townsend index, blood pressure (DBP and SBP), obesity (BMI, body fat percentage, WHR), lipid medications, T2D, and medications affecting renal excretion.
Abbreviations: HR, hazard ratio; OR, odds ratio.

Figure S2.

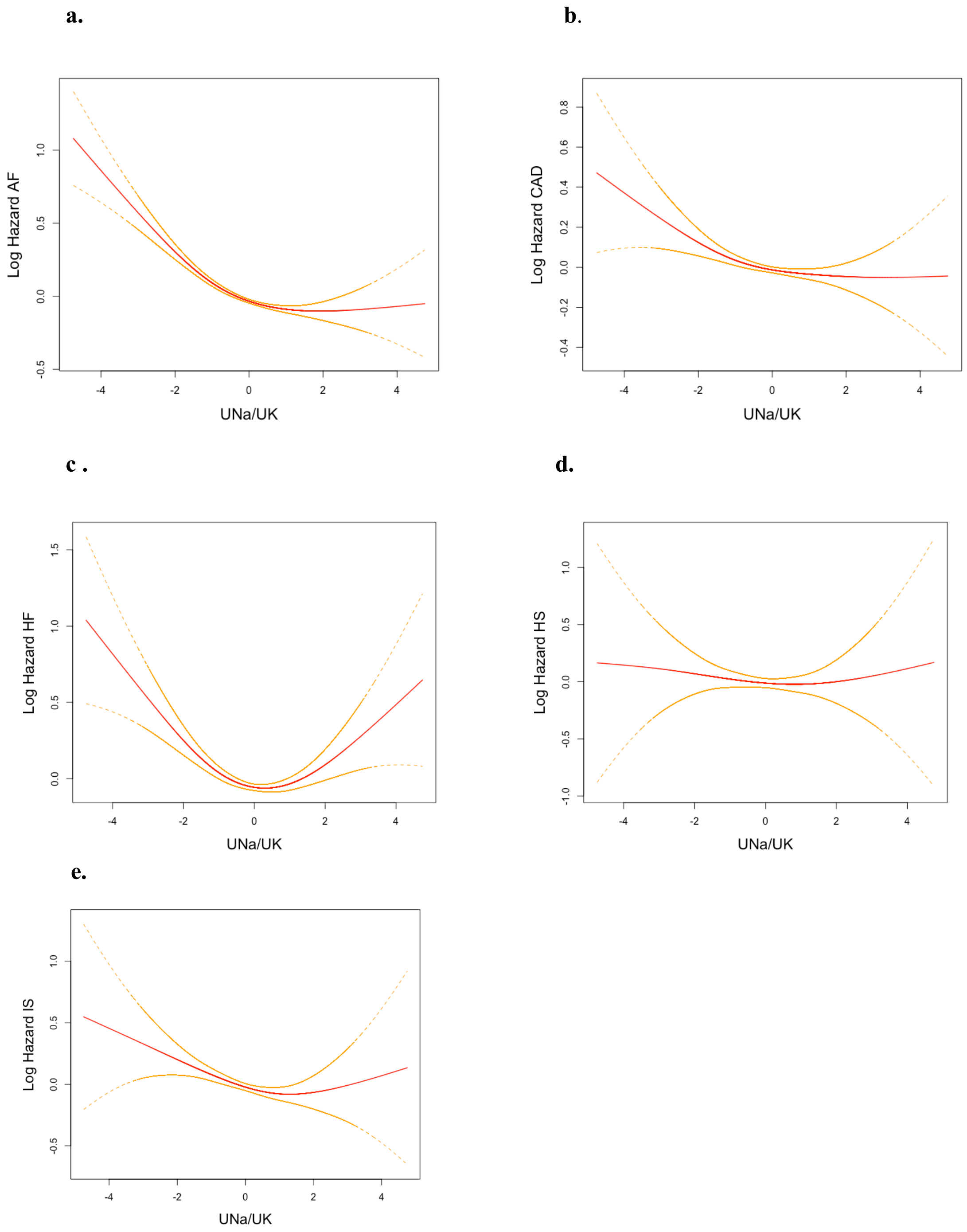

Relations of the urinary sodium to potassium ratio (UNa/UK) with a. atrial fibrillation (AF); b. coronary artery disease (CAD); c. heart failure (HF); d. hemorrhagic stroke (HS); e. ischemic stroke (IS). Lines are based on a regression spline of Cox proportional hazards.

Figure S3.

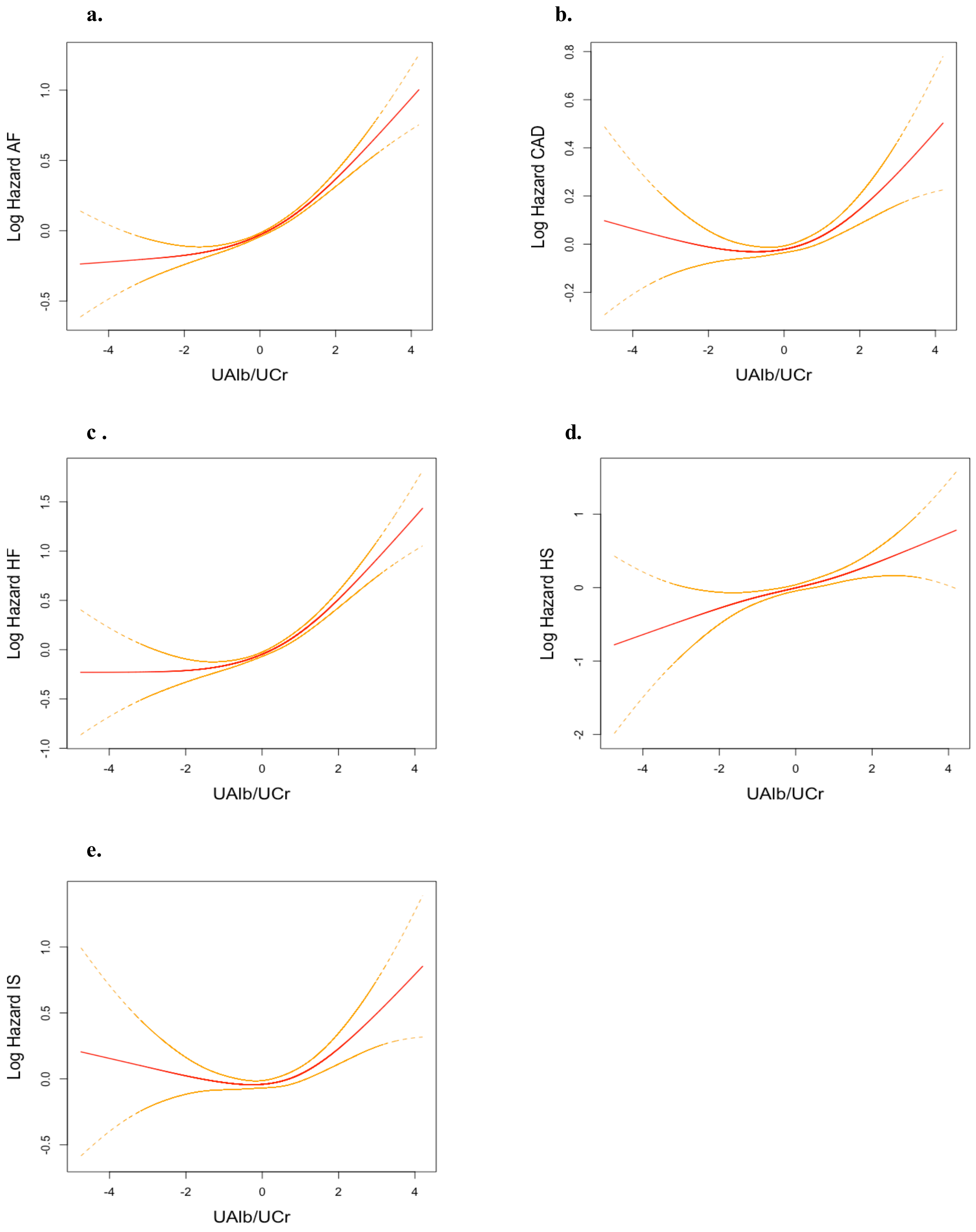

Relations of the urinary albumin to creatinine ratio (UAlb/UCr) with a. atrial fibrillation (AF); b. coronary artery disease (CAD); c. heart failure (HF); d. hemorrhagic stroke (HS); e. ischemic stroke (IS). Lines are based on a regression spline of Cox proportional hazards.

Figure S4.

a.

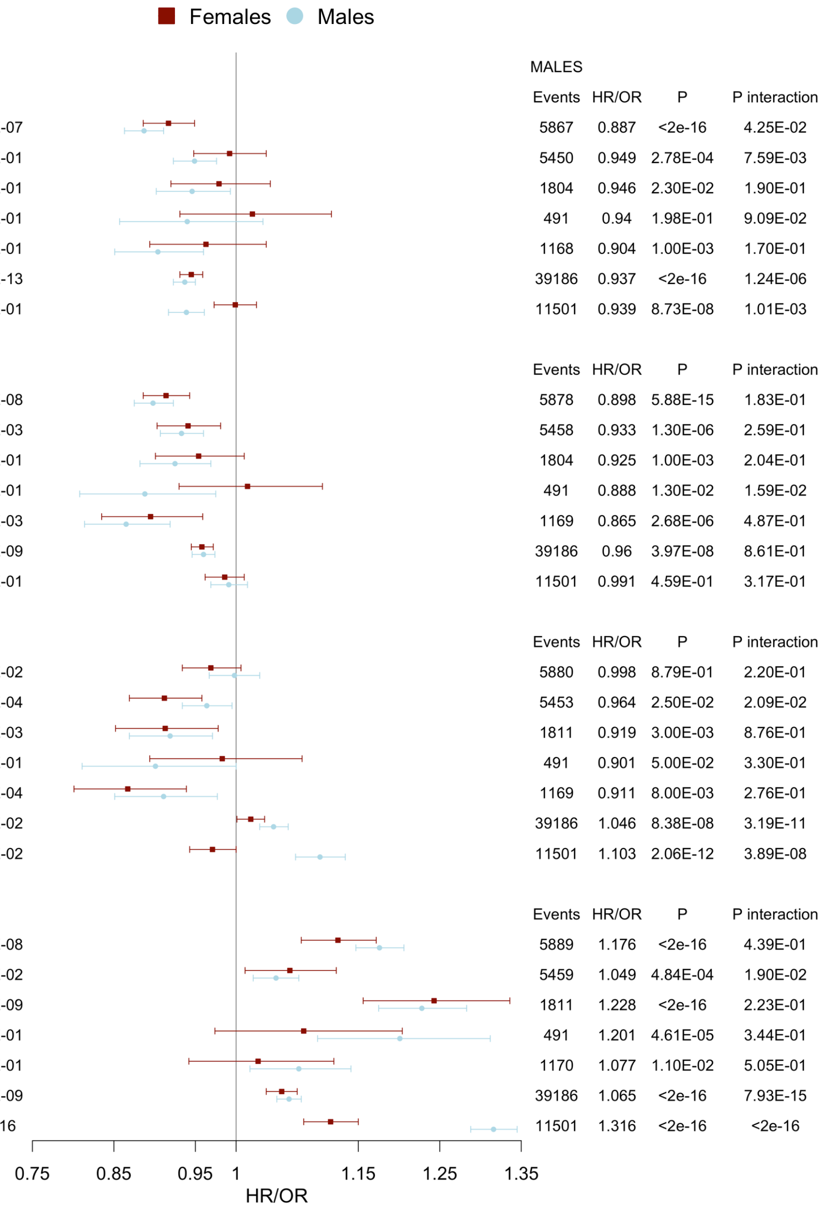

		FEMALES Events	HR/OR	P	MALES Events	HR/OR	P	P interaction
UNa/UK	AF	3329	0.917	6.22E-07	5867	0.887	<2e-16	4.25E-02
	CAD	1925	0.992	7.15E-01	5450	0.949	2.78E-04	7.59E-03
	HF	971	0.979	5.13E-01	1804	0.946	2.30E-02	1.90E-01
	HS	487	1.02	6.75E-01	491	0.94	1.98E-01	9.09E-02
	IS	720	0.963	3.18E-01	1168	0.904	1.00E-03	1.70E-01
	LIPIDS	28711	0.945	3.25E-13	39186	0.937	<2e-16	1.24E-06
	T2D	8375	0.999	9.37E-01	11501	0.939	8.73E-08	1.01E-03
		Events	HR/OR	P	Events	HR/OR	P	P interaction
UNa/UCr	AF	3341	0.914	2.30E-08	5878	0.898	5.88E-15	1.83E-01
	CAD	1929	0.941	5.00E-03	5458	0.933	1.30E-06	2.59E-01
	HF	972	0.954	1.06E-01	1804	0.925	1.00E-03	2.04E-01
	HS	487	1.014	7.45E-01	491	0.888	1.30E-02	1.59E-02
	IS	719	0.895	2.00E-03	1169	0.865	2.68E-06	4.87E-01
	LIPIDS	28711	0.958	3.39E-09	39186	0.96	3.97E-08	8.61E-01
	T2D	8375	0.986	2.56E-01	11501	0.991	4.59E-01	3.17E-01
		Events	HR/OR	P	Events	HR/OR	P	P interaction
UK/UCr	AF	3349	0.969	9.70E-02	5880	0.998	8.79E-01	2.20E-01
	CAD	1932	0.912	2.23E-04	5453	0.964	2.50E-02	2.09E-02
	HF	978	0.913	9.00E-03	1811	0.919	3.00E-03	8.76E-01
	HS	488	0.983	7.18E-01	491	0.901	5.00E-02	3.30E-01
	IS	725	0.867	4.14E-04	1169	0.911	8.00E-03	2.76E-01
	LIPIDS	28711	1.018	3.71E-02	39186	1.046	8.38E-08	3.19E-11
	T2D	8375	0.971	5.39E-02	11501	1.103	2.06E-12	3.89E-08
		Events	HR/OR	P	Events	HR/OR	P	P interaction
UAlb/Cr	AF	3362	1.125	1.28E-08	5889	1.176	<2e-16	4.39E-01
	CAD	1938	1.066	1.80E-02	5459	1.049	4.84E-04	1.90E-02
	HF	980	1.243	4.40E-09	1811	1.228	<2e-16	2.23E-01
	HS	488	1.083	1.42E-01	491	1.201	4.61E-05	3.44E-01
	IS	725	1.027	5.44E-01	1170	1.077	1.10E-02	5.05E-01
	LIPIDS	28711	1.056	4.14E-09	39186	1.065	<2e-16	7.93E-15
	T2D	8375	1.116	<2e-16	11501	1.316	<2e-16	<2e-16

b.

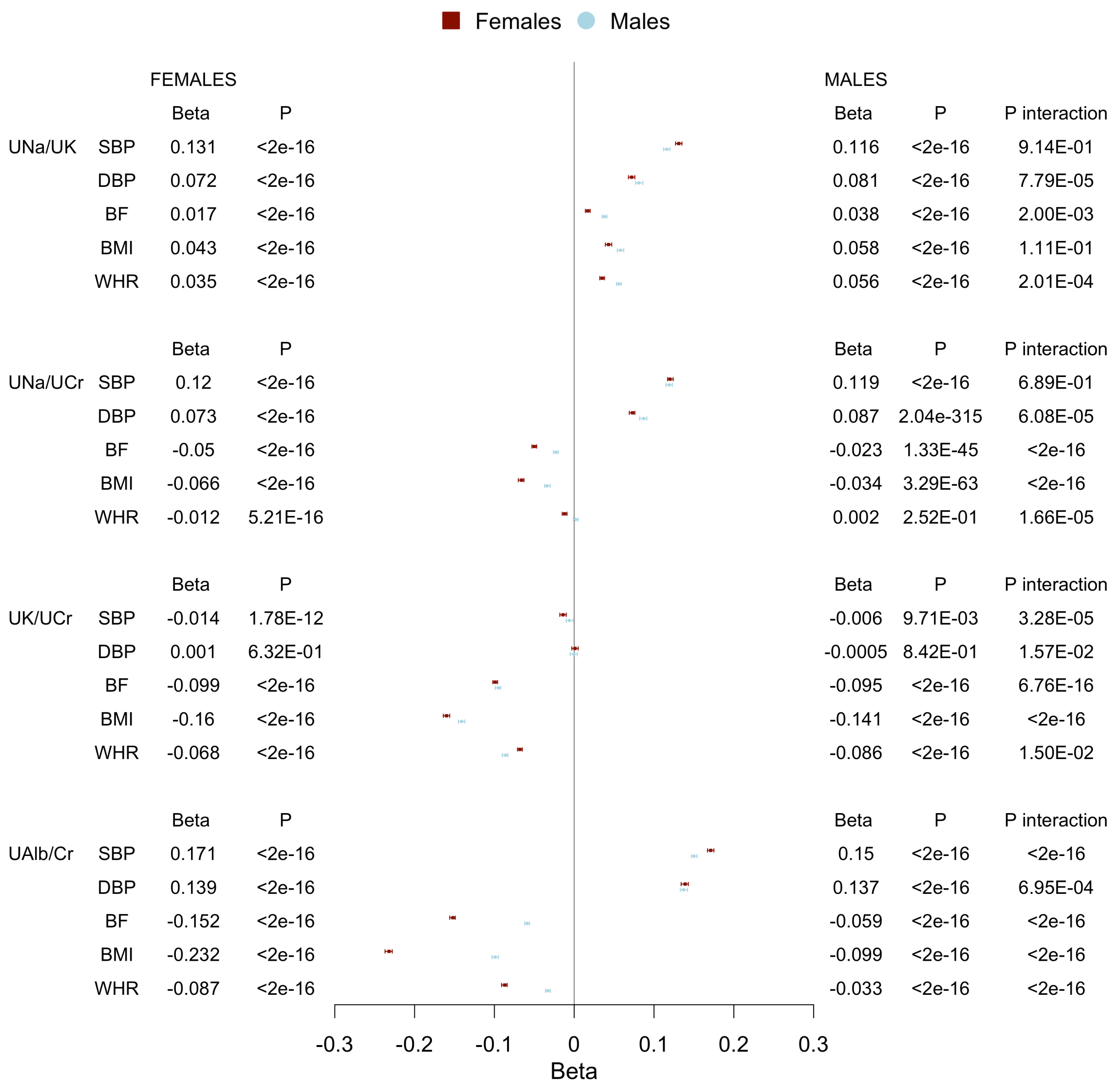

Association results (BETA/HR/OR) of urinary sodium to potassium ratio (Na/UK), urinary sodium to creatinine ratio (UNa/UCr), urinary potassium to creatinine ratio (UK/UCr) and urinary albumin to creatinine ratio (UAlb/UCr) stratified by sex with cardiovascular outcomes in UK Biobank using multivariable-adjusted linear and logistic regression, and multivariable-adjusted Cox proportional hazards models in main analyses. a. Binary outcomes: atrial fibrillation (AF); coronary artery disease (CAD); heart failure (HF); hemorrhagic and ischemic stroke (HS and IS, respectively); lipid medications (LIP) and type 2

diabetes (T2D). **b.** Continuous outcomes: systolic and diastolic blood pressure (SBP and DBP, respectively), body fat percentage (BF); body mass index (BMI), waist-to-hip ratio (WHR).

Sex-stratified analysis (N males= 212,534; N females= 265,777) performed in the main cohort excluding participants with diagnosis indicating decreased kidney function (N= 7,221) and/or cardiovascular disease at baseline (N= 17,087). The betas from linear regression represent SD change in outcome variable per SD change in UNa/UK. Model adjustment: sex, age, region of the UK Biobank assessment center, ethnicity, smoking, alcohol, physical activity, Townsend index, blood pressure (DBP and SBP), obesity (BMI, body fat percentage, WHR), lipid medications, T2D, and medications affecting renal excretion.
Abbreviations: HR, hazard ratio; OR, odds ratio.

Figure S5.

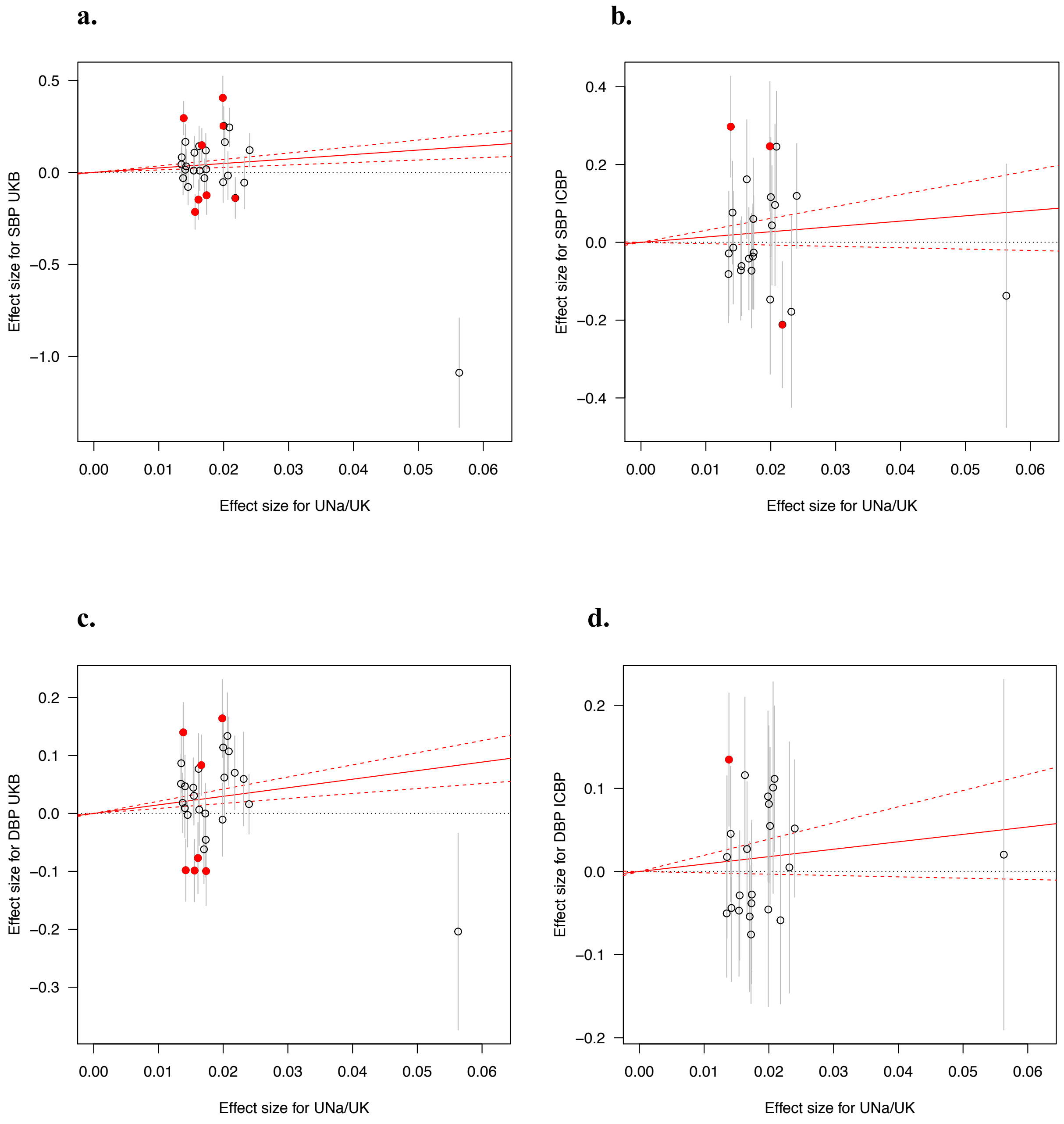
a.
b.
c.
d.
Effect size for SBP UKB
Effect size for SBP ICBP
Effect size for DBP UKB
Effect size for DBP ICBP
Effect size for UNa/UK
0.5
0.0
−0.5
−1.0
0.4
0.2
−0.2
−0.4
0.1
−0.1
−0.3
0.00
0.01
0.02
0.03
0.04
0.05
0.06

e.

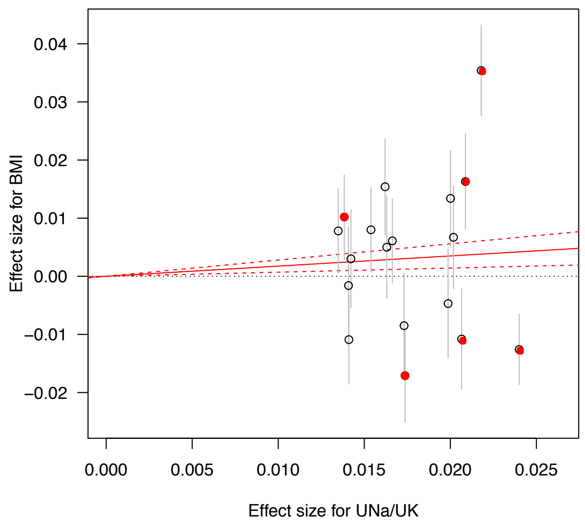

f.

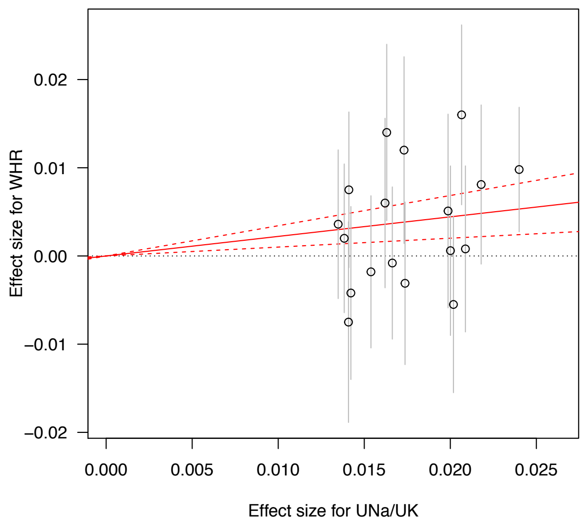

Effects of genetic variants on urinary sodium to potassium ratio (UNa/UK) and risk of a. systolic blood pressure in UK Biobank (SBP UKB); b. systolic blood pressure from International Consortium of Blood Pressure (SBP ICBP); c. diastolic blood pressure in UK Biobank (DBP UKB); d. diastolic blood pressure from International Consortium of Blood Pressure (DBP ICBP); e. body mass index (BMI); f. waist-to-hip ratio (WHR). Variants showing significant heterogeneity are highlighted in red.

Figure S6.

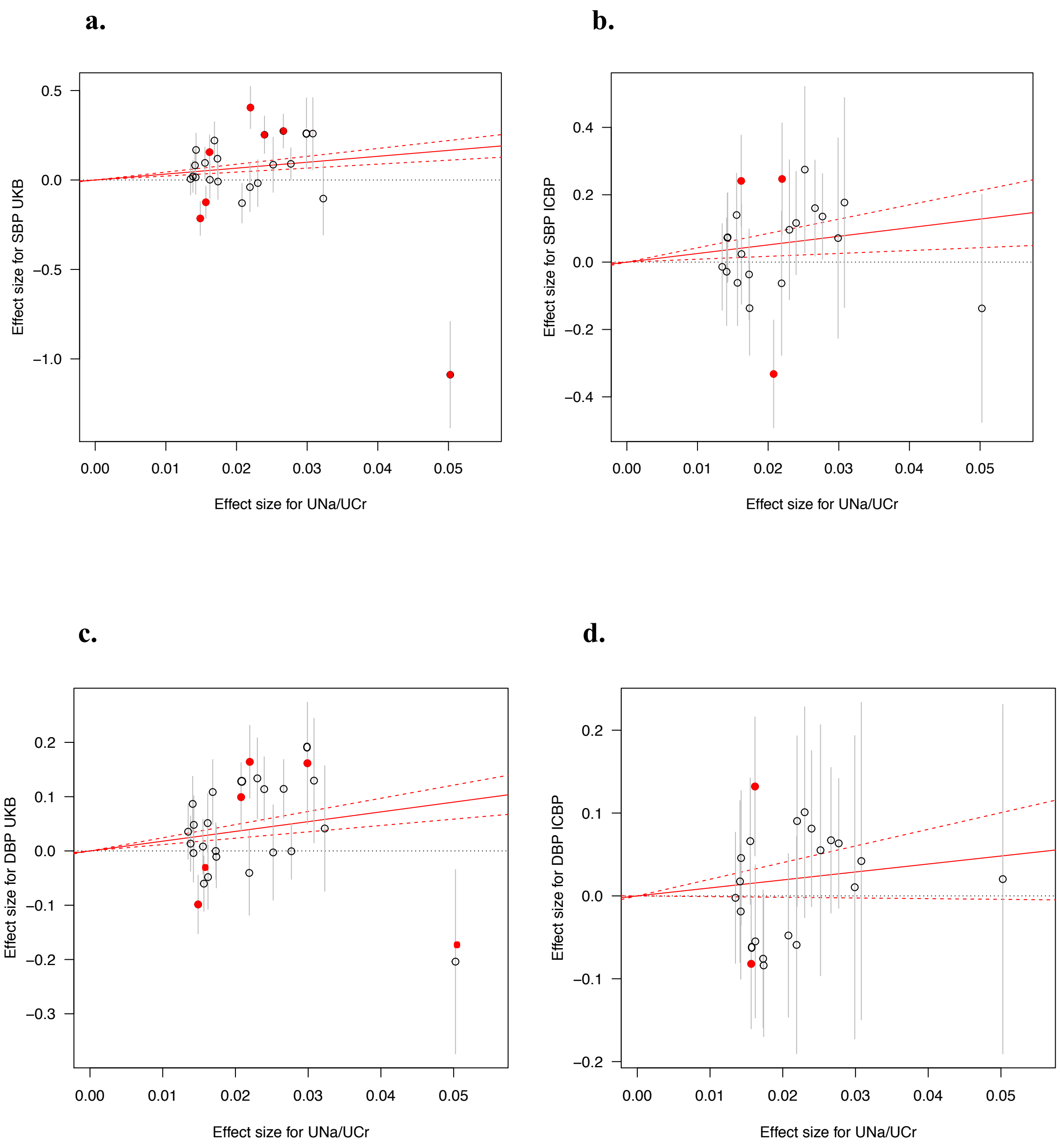
a.
b.
c.
d.
Effect size for SBP UKB
Effect size for SBP ICBP
Effect size for DBP UKB
Effect size for DBP ICBP
Effect size for UNa/UCr
0.00
0.01
0.02
0.03
0.04
0.05
0.5
0.0
−0.5
−1.0
0.4
0.2
−0.2
−0.4
0.1
−0.1
−0.3

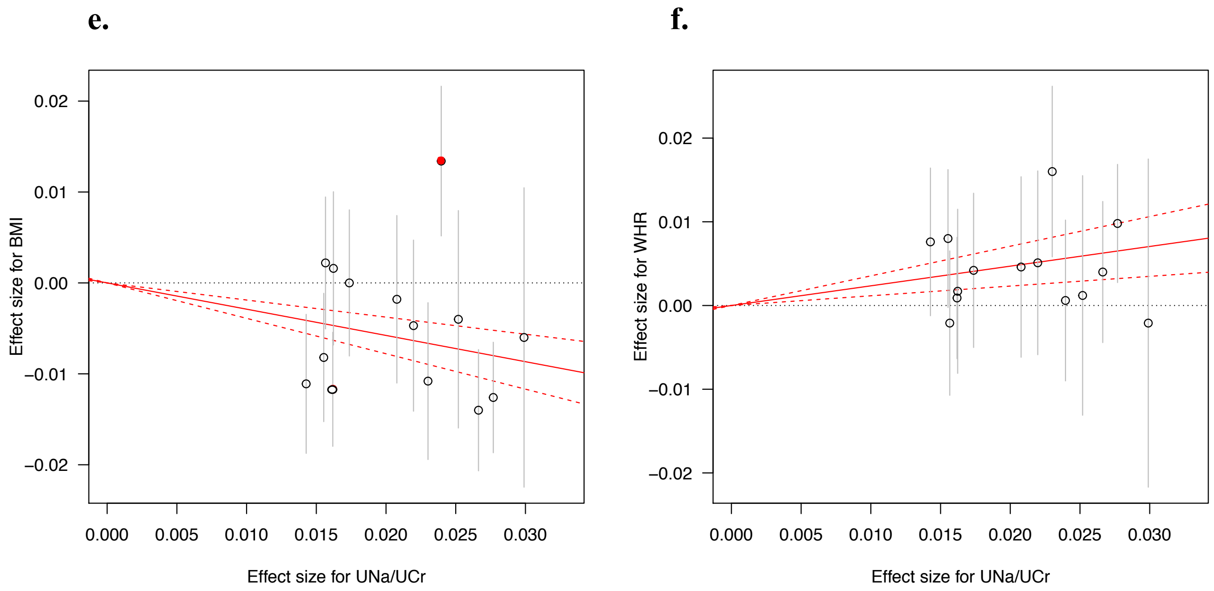

Effects of genetic variants on urinary sodium to creatinine ratio (UNa/UCr) and risk of a. systolic blood pressure in UK Biobank (SBP UKB); b. systolic blood pressure from International Consortium of Blood Pressure (SBP ICBP); c. diastolic blood pressure in UK Biobank (DBP UKB); d. diastolic blood pressure from International Consortium of Blood Pressure (DBP ICBP); e. body mass index (BMI); f. waist-to-hip ratio (WHR). Variants showing significant heterogeneity are highlighted in red.

Figure S7.

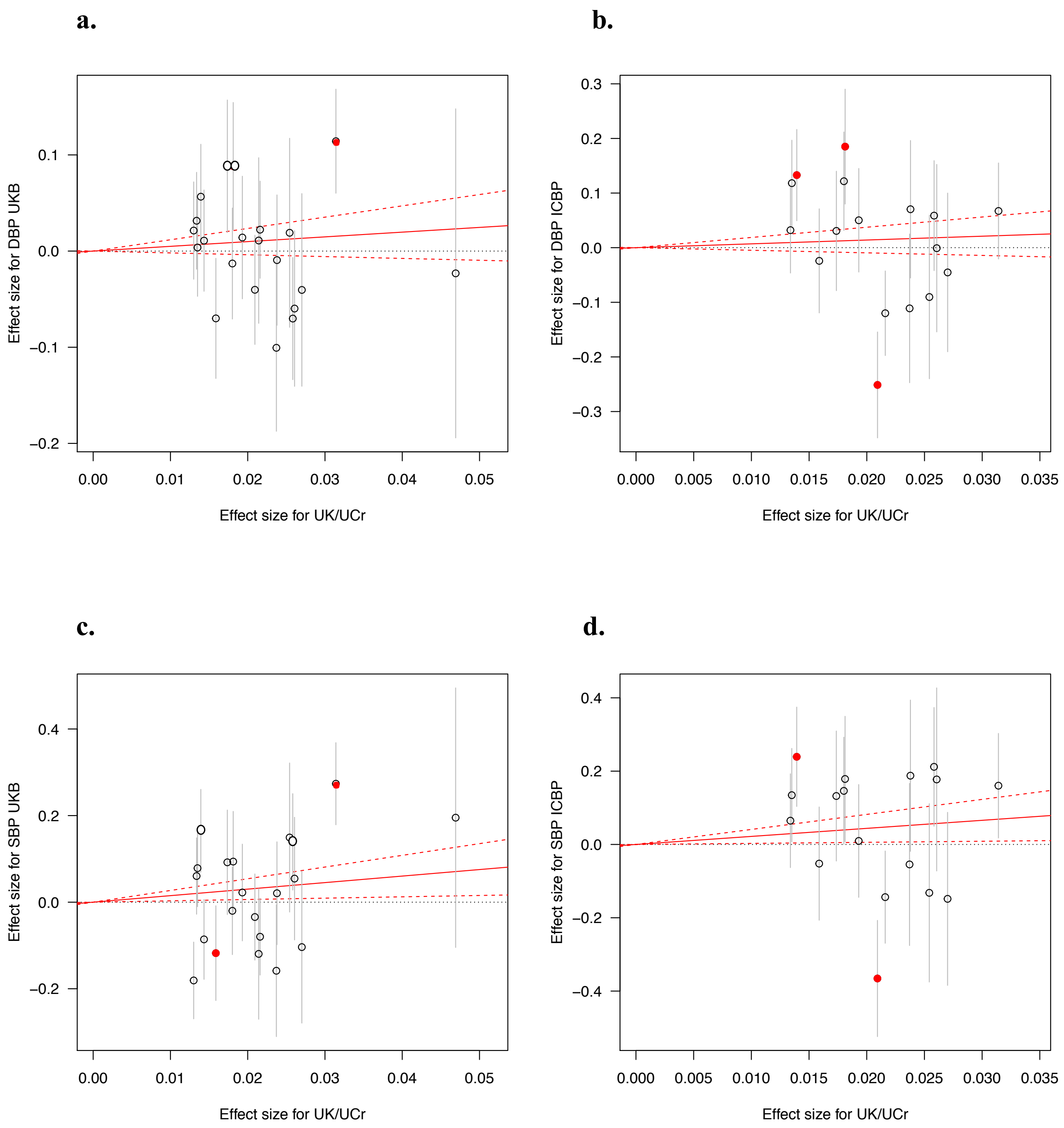
a.
Effect size for DBP UKB
0.1
0.0
−0.1
−0.2
0.00
0.01
0.02
0.03
0.04
0.05
Effect size for UK/UCr
b.
Effect size for DBP ICBP
0.3
0.2
0.1
0.0
−0.1
−0.2
−0.3
0.000
0.005
0.010
0.015
0.020
0.025
0.030
0.035
Effect size for UK/UCr
c.
Effect size for SBP UKB
0.4
0.2
0.0
−0.2
0.00
0.01
0.02
0.03
0.04
0.05
Effect size for UK/UCr
d.
Effect size for SBP ICBP
0.4
0.2
0.0
−0.2
−0.4
0.000
0.005
0.010
0.015
0.020
0.025
0.030
0.035
Effect size for UK/UCr

e. **f.**

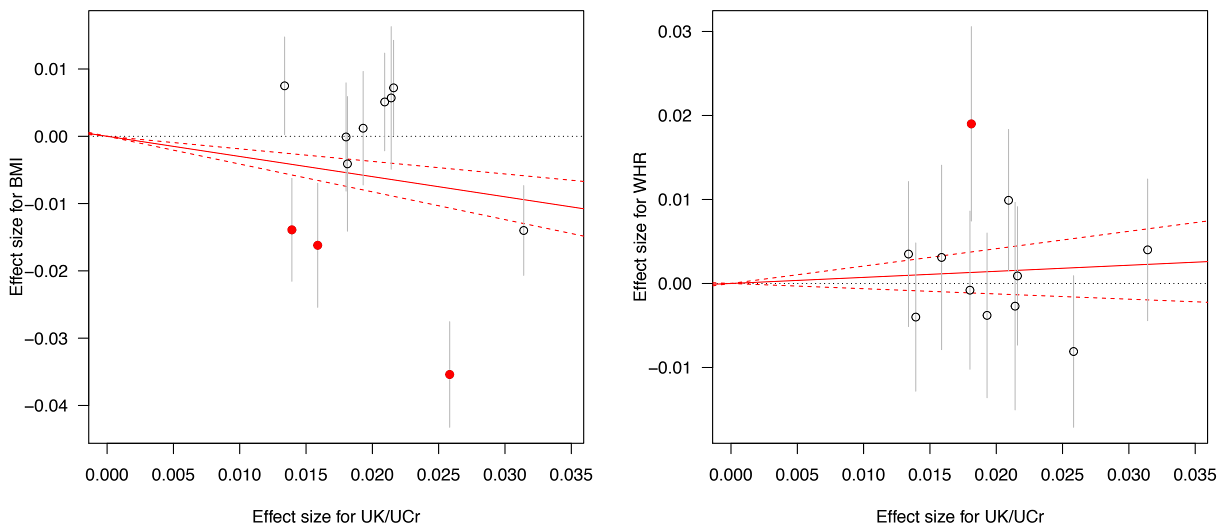

Effects of genetic variants on urinary potassium to creatinine ratio (UK/UCr) and risk of a. systolic blood pressure in UK Biobank (SBP UKB); b. systolic blood pressure from International Consortium of Blood Pressure (SBP ICBP); c. diastolic blood pressure in UK Biobank (DBP UKB); d. diastolic blood pressure from International Consortium of Blood Pressure (DBP ICBP); e. body mass index (BMI); f. waist-to-hip ratio (WHR). Variants showing significant heterogeneity are highlighted in red.

Figure S8.

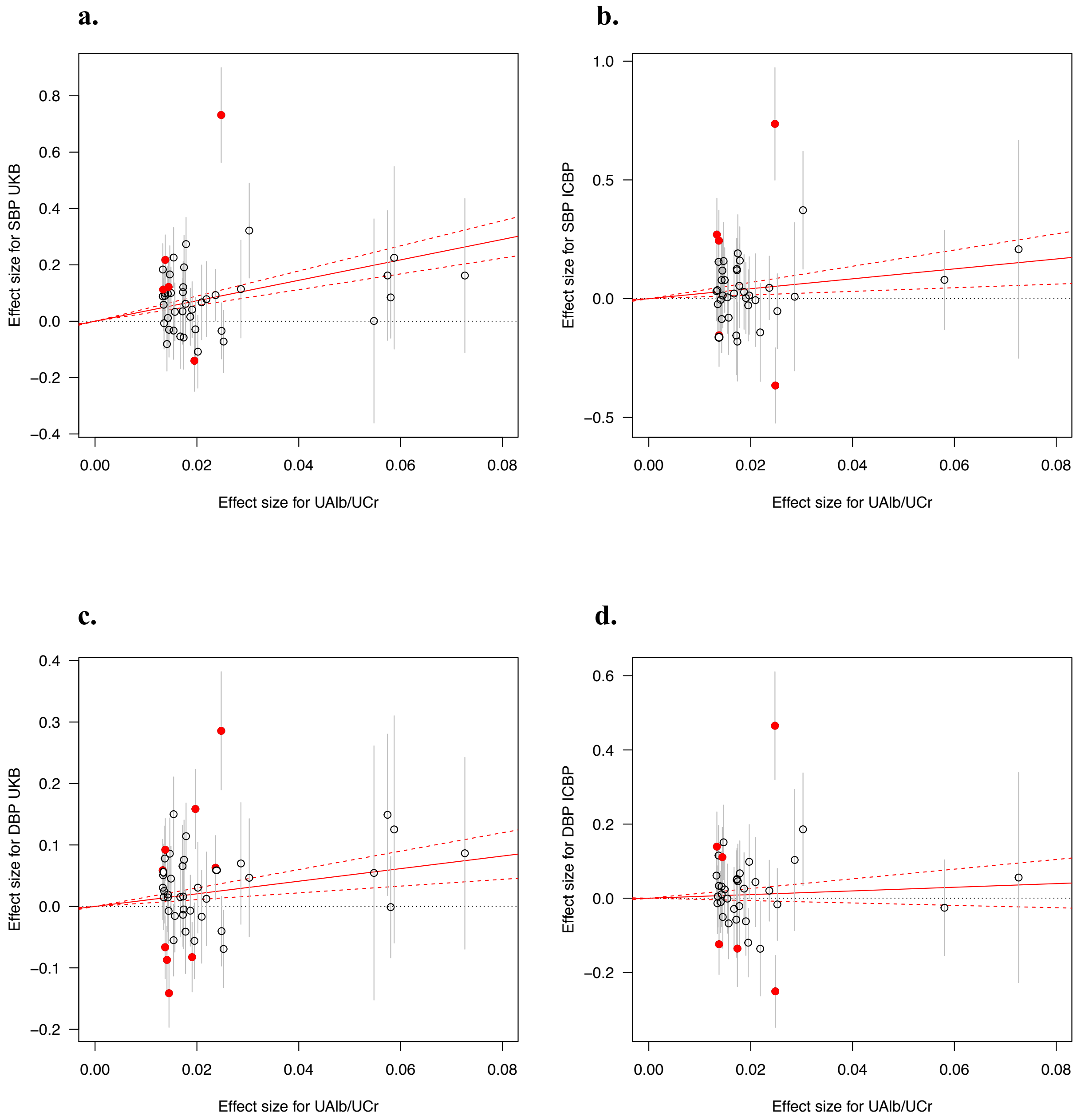
a.
b.
c.
d.
Effect size for SBP UKB
Effect size for SBP ICBP
Effect size for DBP UKB
Effect size for DBP ICBP
Effect size for UAlb/UCr
0.00
0.02
0.04
0.06
0.08

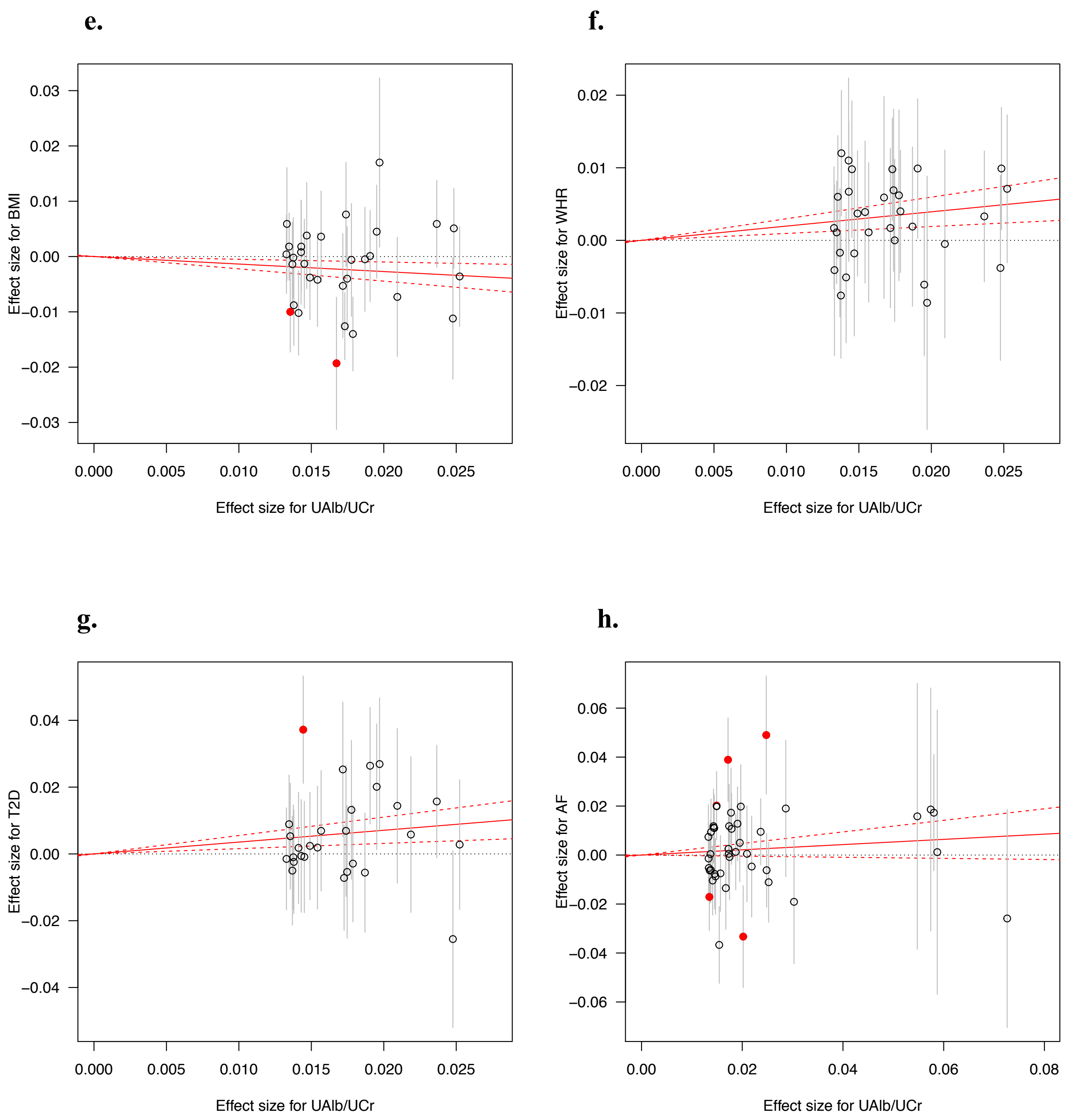

Effects of genetic variants on urinary albumin to creatinine ratio (UAlb/UCr) and risk of a. systolic blood pressure in UK Biobank (SBP UKB); b. systolic blood pressure from International Consortium of Blood Pressure (SBP ICBP); c. diastolic blood pressure in UK Biobank (DBP UKB); d. diastolic blood pressure from International Consortium of Blood Pressure (DBP ICBP); e. body mass index (BMI); f. waist-to-hip ratio (WHR); g. type 2 diabetes (T2D); h. atrial fibrillation (AF). Variants showing significant heterogeneity are highlighted in red.
